# Usability of a digital mindfulness training program for smoking cessation: A mixed-method single-center pilot study protocol (HowToMind)

**DOI:** 10.1371/journal.pone.0318686

**Published:** 2025-02-20

**Authors:** Anastasia Demina, Agnès Soudry-Faure, Benjamin Petit, Nicolas Meunier-Beillard, Benoit Trojak

**Affiliations:** 1 Addiction Medicine Department, Dijon Bourgogne University Hospital, Dijon, France; 2 INSERM U1093, CAPS, Université de Bourgogne, UFR STAPS, Dijon, France; 3 Unité de Soutien Méthodologique à la Recherche DRCI Dijon Bourgogne University Hospital, Dijon, France; PLOS: Public Library of Science, UNITED KINGDOM OF GREAT BRITAIN AND NORTHERN IRELAND

## Abstract

**Background:**

Tobacco use is one of the leading causes of preventable disease and death worldwide. Despite the availability of evidence-based pharmacological treatments, only a small number of individuals with tobacco use disorder achieve long-term abstinence after smoking cessation. This highlights the need to enhance existing interventions. In this protocol, we describe our single-center mixed-method trial, HowToMind, conducted in Dijon, France. This trial aims to investigate the usability and acceptability of a digital mindfulness-based intervention designed to complement standard smoking cessation treatment to potentiate its effects.

**Methods:**

We will include 60 adults seeking treatment for tobacco use disorder, as defined by DSM-5 criteria, who wish to quit smoking and own a smartphone. All participants will receive a combination of transdermal and oral nicotine replacement therapy and will be introduced to an eHealth app that provides a digital equivalent of an 8-week mindfulness training program. The acceptability of the initial version of our app will be assessed based on usage frequency, and usability will be evaluated using the Mobile App Rating Scale (French version). A participatory approach will be employed through focus groups conducted at the end of the 8 weeks of app use, aimed at co-constructing the final version of the app based on participant feedback.

**Discussion:**

Our pilot mixed-method trial seeks to explore the usability and acceptability of our app, making necessary adjustments to its content and functionality based on participant feedback before its implementation in a large randomized controlled trial assessing the app’s potential to enhance the effects of standard treatment.

**Trial registration:**

ClinicalTrials.gov, NCT06500117.

## Background

Tobacco use is one of the leading causes of preventable disease and death worldwide [1]. Globally, more than 8 million deaths are caused each year by tobacco, and public health estimates suggest that if current levels of smoking continue, 1 billion deaths will be directly related to tobacco in the 21st century [2, 3].

Several therapeutic approaches, including nicotine replacement therapy (NRT), varenicline, or buproprion, have demonstrated a significant impact on smoking cessation [4]. A recent Cochrane review shows that NRT increases the rates of smoking cessation success by 50 to 60% with a relative risk (RR) of 1.55 (1.49; 1.61) [5]. However, given that only 3 to 5% of individuals remain abstinent 6 to 12 months after a smoking cessation attempt, NRT would increase the total success rates by only 2 to 3% [5, 6]. Other therapeutic options such as varenicline or bupropion, which reduce craving and withdrawal symptoms, are effective in long-term smoking cessation success, with RR of 2.88 (2.10; 3.96) for varenicline compared to placebo and RR of 1.77 (1.28; 2.46) for bupropion compared to placebo [7]. However, their use is often undermined by their side effects. Varenicline is known to provoke nausea, vomiting, insomnia or abnormal dreams, and bupropion can cause insomnia, agitation, dry mouth or headaches [8, 9].

Neurobiological research highlights the importance of prefrontal control in smoking cessation strategies. More specifically, due to the lessening of the prefrontal control over smoking behavior and the resulting executive disruption, smoking becomes automatized and compulsive in response to the negative affect, stress and craving [10]. Interventions reinforcing prefrontal control and alleviating craving could be a valuable addition to the existing therapeutic options, potentiating their efficacy on smoking cessation and abstinence.

Mindfulness training programs or mindfulness-based interventions (MBIs) are evidence-based interventions teaching individuals to engage in a direct and non-judgmental experience of the present moment through mindfulness meditation practice [11]. The neurobehavioral effects of MBIs are mediated by the mechanisms of attentional allocation, translating into indicators of effectiveness in depression, stress reduction, and chronic pain management [12, 13]. Not only do MBIs facilitate attention allocation focused on the present moment, with a reinforcement of decision-making mechanisms not in response to impulsivity, but in full awareness, but they also aim to facilitate acceptance and tolerance of discomfort, which could reduce the need to smoke to alleviate cravings and negative affect [11, 14]. MBIs appear to have a significant effect on craving and may influence a variety of other potential therapeutic targets of interest in addiction, such as stress, reactivity to cues, attentional biases, and psychological flexibility [15, 16]. Finally, MBIs have no significant adverse effects, making them highly acceptable [17].

Conventional MBIs show promising results for smoking cessation, as highlighted by a recent meta-analysis in which MBIs had an estimated RR of 1.88 to 95% [1.04; 3.40] for 7-day point abstinence 17 to 24 weeks after the intervention [18]. However, conventional MBIs may be unfeasible for many patients because of the need to attend several weekly group sessions in a row, and the cost, which is often not covered by health insurance. To overcome the practical issues of conventional MBIs, several smartphone applications (apps) have been developed. However, they often lack the rich, intensive, and progressive training process of a standard 8-week program. Importantly, if an app is meant to be used in clinical settings as an e-Health tool, it must satisfy safety, quality of information, patient acceptability and usability or ease of use requirements [19]. With these requirements in mind, we created the first mindfulness app for smoking cessation that allows patients to follow a complete 8-week MBI recreating as closely as possible the conditions of a conventional MBI. Indeed, the app is implemented to a French EXOLIS platform which is certified for hosting health data. The quality of the information is supported by the fact that addiction health professionals trained in MBIs created its content. However, there is a need for a thorough evaluation of its acceptability and usability by the patients it was created for.

In this protocol, we describe our single-center (CHU Dijon Bourgogne, France) mixed-method trial HowToMind, which aims to investigate our app’s usability and acceptability in a population of smokers seeking smoking cessation with standard treatment, and to co-construct the final version of the app with participant feedback. Indeed, incorporating patient input is essential when developing a new health intervention prior to its large-scale implementation. This promotes user empowerment and active participation, which can enhance the acceptability and appropriateness of the intervention for the intended population [20, 21].

## Methods

This study was registered on ClinicalTrials.gov under NCT number NCT06500117.

### Study population

We will include adults consulting for tobacco use disorder according to DSM-5 criteria, wishing to quit smoking and owning a smartphone. We will not include individuals with cognitive impairment that would prevent mindfulness training or suffering from an acute or non-stabilized psychiatric or somatic disorder.

A total of 60 participants will be included. This number is justified by the fact that in addition to acceptability and usability evaluation, we are planning to conduct three focus groups with 6 to 8 participants in each. Given the potential loss to follow-up and scheduling challenges for the focus groups, this sample size seems to correspond well to the chosen methodology. It also conforms to sample sizes commonly used in pilot studies.

We expect a high level of feasibility for our recruitment, facilitated by the mild intensity of our intervention. The principal investigator is a practicing addiction physician trained in MBIs and with extensive experience in clinical research in tobacco use disorder. The inclusion period will last 21 months. The duration of the follow-up will be set at 3 months. The study will therefore last 24 months.

### Intervention

The intervention will be implemented in two phases. The first, guided learning phase (8 weeks) will consist of mindfulness training via the initial version of our smartphone app in addition to the standard treatment (NRT). The app features eight thematic video modules that progressively introduce elements of mindfulness meditation in relation to smoking cessation (**Table 1**). The key messages of the videos will be available in form of PDF documents each week. For each module, audio sessions of varying durations will be available to facilitate daily practice (body scan, mindful movement, breathing, etc.). Modules will target specific elements of tobacco use disorder such as craving and automatic behavior, as well as more general elements such as emotion regulation, self-judgment, and acceptance. During this guided learning phase, a new module will be made available each week. The participants will be instructed to use the app at least once a day.

**Table 1:** Digital mindfulness training program description.

Module	Details	Content
**Week one: Automatic behavior**	Concepts of mindfulness and automatic behavior, exercises focused on facilitation of mindful awareness of different parts of everyday life	For each module:
		Video document (15 minutes),
**Week two: Awareness of bodily sensations**	Awareness of bodily sensations, introduction to bodyscan exercises	PDF document with key messages,
**Week three: Emotional regulation**	Patterns of emotional dysregulation and their role in smoking behavior, focused attention training	3 audio documents for daily practice
**Week four: Craving**	Craving phenomenon and ways to cope, mindfulness applied to craving in form of non-judgmental awareness and observation	
**Week five: Shame, guilt, judgment**	Role of shame and guilt in smoking behavior and relapse, non-judgmental observation of these phenomena	
**Week six: Acceptance**	Acceptance as an active and intentional practice, focused attention and observing mental constructs arising during practice	
**Week seven: Relapse**	Noticing first signs of relapse, observing self-judgment	
**Week eight: Creating one’s own practice**	Guidance to integrate mindfulness to everyday life, bonus sessions	

The second phase of the intervention, or individual practice phase, will facilitate the transition to an autonomous mindfulness practice in participants’ daily life. Thus, after eight weeks of learning, the participants will be able to access all of the app’s resources as part of their own personal practice for four weeks. They will be instructed to use the app’s resources as needed.

### Associated treatment

All participants will be prescribed a combination of transdermal and oral NRT, which is a standard treatment for smoking cessation. The NRT dosages will depend on the patient’s clinical assessment, thus replicating the real-world conditions of NRT treatment. Patients will be provided with post-trial care for smoking cessation if needed.

### Primary outcomes

The acceptability of the initial version of our app will be assessed by the regularity of its use. Participants who use the app at least 4 times a week for 8 weeks will be defined as active users, those who use it 1 to 3 times a week as occasional users, and those who stop using the app as users who have discontinued the intervention.

Usability will be assessed with the Mobile App Rating Scale, French version (MARS-F) after 8 weeks of using the app [22]. The MARS-F Usability Scale is composed of 23 items scored from 1 to 5, and it explores four objective domains (engagement, functionality, aesthetics, information quality) leading to a score between 0 and 95 and a subjective domain at 20 points. Usability will be considered good if at least 75% of users assign a MARS-F score of at least 85 out of 115.

A participatory approach will be deployed in form of focus groups which will be set up at the end of the 8 weeks of app use. Active users, occasional users, and those who have abandoned the use of the app will participate to provide their perceptions and experiences, and to collectively establish recommendations for the necessary changes to implement to the initial version of the app. The knowledge of acceptability and usability of the app, as well as the study of motivations and obstacles to its use, will inform us about the needs of the participants in terms of the functioning of the app and will allow us to make the necessary adjustments before implementing the final version on a larger scale.

### Secondary outcomes

The change in cigarette consumption between baseline and 4 weeks, 8 weeks and 12 weeks will be measured using a smoking diary. A CO test will be performed at baseline, 4 weeks, 8 weeks and 12 weeks. Smoking abstinence will be defined as the absence of tobacco use for 7 days prior to the assessment visit. Craving intensity will be measured at baseline, at 4 weeks, 8 weeks, and 12 weeks using a visual analogue scale.

The smoking diary and a craving log will be implemented in the app. Participants will be asked to provide daily logs for their smoking diary (0 = no consumption, 1 = 1 cigarette, etc.) and weekly logs for craving assessment, by evaluating the intensity of their maximum craving over the last seven days (from 1 to 10). Similarly, mindfulness practice diary will be accessible via the app. Participants will be invited to provide a daily report on their mindfulness practice (Yes/No). Participants will receive gentle daily reminders to complete the logs. In case of a non-viewed video, participant will also receive a reminder. In case of non-completion of either the smoking diary, craving or mindfulness practice logs, or if the video content is not viewed by the participant, the Principal Investigator will receive alerts and will contact the participant to offer assistance.

### Study timeline

Participants will be recruited through physician’s referral after communication in local press and in addiction treatment facilities. An investigator physician will identify eligible participants and obtain their free and informed verbal consent documented by the investigator using the consent form (**S1 Document**). During the inclusion visit, the investigator physician will present the app and will instruct the participant how to download it. The first connection to the app will take place during this visit to explain how it works and to enter the participant’s inclusion number. After the inclusion visit, participants will engage in smoking cessation using NRT and the app. Inclusion visits will be grouped into three blocks of 20 patients for the constitution and conduct of focus groups.

Participants will benefit from follow-up visits 4 and 8 weeks after inclusion. Focus groups will be carried out shortly after the 8-week visit to avoid memory bias. The following themes will be addressed during the focus groups: knowledge/representations of e-Health tools, access to the app, general aspect, flexibility and adaptability of use, perceived impact on smoking-related behavior, and recommendations for improvement.

**Fig 1:**
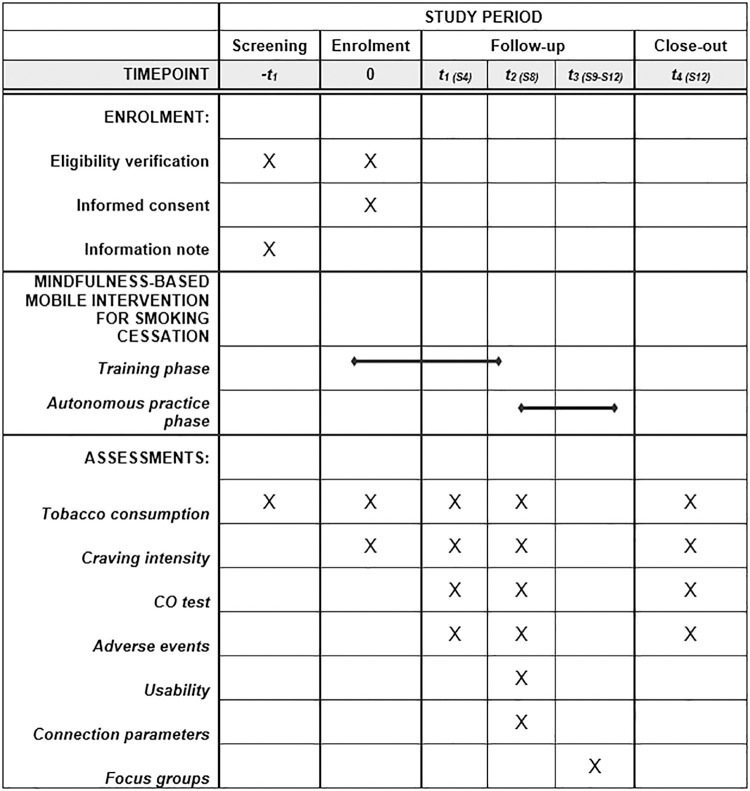
Schedule of enrolment, interventions, and assessments.

The end-of-study visit will occur at 12 weeks post-inclusion. All included individuals will be followed until the end of the study except for patients who withdraw their consent. After analysis and modifications of the app based on participant feedback, we will offer a feedback session to present the results of the study and the new version of the app. The study timeline is represented in **Fig 1**.

At the time of protocol submission, only seven patients had been included. The first participant was enrolled on July 1, 2024. The estimated completion date for data collection is September 15, 2025. Analyses are scheduled to begin in October 2025, with results expected by December 2025.

### Data management

Ownership and use of the data will be exclusive to the study sponsor. The data collected during medical visits are those traditionally collected in the context of smoking cessation management (age of smoking onset, number of cigarettes per day, history of smoking cessation and therapeutic means, use of e-cigarettes, smokers or vaping in the entourage, and CO measurement). Data on the regularity of app use (active user, occasional user, discontinuation of use) will be also collected. These data will be collected in an electronic CRF (e-CRF) created using the CleanWeb software by a data manager. Data hosting via the CleanWEB software is provided by Telemedicine Technologies via its secure web hosting platform. A copy of the extraction file in "csv" format from the frozen database will also be kept on a server of the Dijon Bourgogne University Hospital, secured by a password by the data manager.

Data on smoking and craving intensity (data commonly collected as part of standard care), as well as on regularity of mindfulness practice, will be provided by participants using the app. The qualitative data collected during focus groups will be recorded using a digital dictaphone. They will then be transcribed verbatim while respecting the anonymity of the participants and hosted in a secure manner on the servers of the Dijon Bourgogne University Hospital until they are deleted at the end of the study.

The security, quality and access to study data will be managed by the data manager. Data entries will be validated by dynamic consistency checks implemented during the construction of the database. To validate the consistency of the data, series of queries will be carried out periodically by the data manager. These queries will be repeated iteratively until an error-free database is obtained.

### Statistical analysis

Analyses will be carried out on the frozen database resulting from the reconciliation of the data collected on CleanWEB and EXOLIS. The MARS-F usability score will be calculated for each participant after the 8-week period of use. The score will be presented as a mean and standard deviation with its 95% confidence interval. The percentage of active and occasional users as well as those who have abandoned the use of the app will also be calculated. Secondary analyses will be descriptive and/or exploratory. Quantitative variables will be described as means with their standard deviations, medians, and interquartile ranges. SAS Software Version 9.4 will be used for quantitative analyses.

Qualitative data will be processed by thematic analysis [23]. It offers a valuable qualitative approach for applied research, emphasizing the inductive process to establish a strong link between field data and themes. The six steps of thematic analysis include familiarization with the data, generating initial codes, searching for themes, reviewing themes, defining and naming themes, and producing the report. Multidisciplinary sessions for coding triangulation —including addiction physicians, public health researchers, and epidemiologists—will occur during the constitution of the descriptive analysis framework and again during the creation of the thematic tree (axial coding). NVivo software will be used for assisting coding steps and facilitate the categorization and grouping of encoded data.

### Regulatory aspects

The planning and conduct of this study are governed by French law (Law No. 2012-300 of 5 March 2012 on research involving human beings, amended by Ordinance No. 2016-800 of 16 June 2016 and its implementing decrees) [24]. The study will be conducted in accordance with the ethical principles of the Declaration of Helsinki and the recommendations of Good Clinical Practice [25, 26]. The computer file used to carry out this research will be subject to application of the "Data Protection Act", *Law No*. *78-17 of 6 January 1978 relating to data processing*, *files and freedoms as amended and the General Data Protection Regulation* [27, 28].

At the end of the study, all documents related to the study (including copies of case reports) will be archived at the study site. The results of the study, whatever they may be, will be submitted for publication. Any publication or communication (oral or written) will be decided by mutual agreement between the investigators and will comply with the international recommendations of the *International Committee of Medical Journal Editors* [29].

## Discussion

This study protocol aims to describe the methodological choices and rationale for our mixed-method trial investigating usability and acceptability of a digital mindfulness-based intervention. An important part of the study is based on qualitative methods providing participant feedback and promoting their participation in the development of novel health interventions. This pilot mixed-method trial seeks to explore whether our app is usable and acceptable, and to make the necessary changes in its content and functionality using the participants’ feedback before its implementation in a large randomized controlled trial.

There is a growing place for eHealth technology in public health. App-based eHealth interventions offer accessible, flexible and cost-effective solutions and promote patients’ autonomy and involvement in their health management. In addiction medicine, apps could also provide opportunities to implement motivational elements, such as notifications and reminders. In addition, repeated questionnaires can be integrated into the app to enable real-time tracking of consumption and craving [30].

The theoretical foundations of MBIs are particularly relevant in smoking cessation. Mindfulness practice, through learning the key messages of the 8-week MBI program, could lead to improved craving tolerance and a reduced risk of relapse after smoking cessation. Since traditional in-person MBIs are not always accessible for many smokers, our app-based solution could represent a viable alternative. For our study, foreseeable side effects are those related to smoking cessation (craving, difficulty concentrating, headaches, irritability, fatigue, constipation) and to the use of NRT (skin irritation, abdominal pain, hiccups, gastroesophageal reflux, sleep disturbances, nausea, diarrhea, dizziness). These symptoms will be monitored throughout the duration of the study.

## Supporting information

S1 Document Consent form. (DOCX)

S1 File (DOCX)

S1 Appendix (DOCX)
