# Supplementary material for: Usability of a digital mindfulness training program for smoking cessation: A mixed-method single-center pilot study protocol (HowToMind)
Source: PLoS One. 2025 Feb 20;20(2):e0318686. doi: 10.1371/journal.pone.0318686 (PMC11841885; doi:10.1371/journal.pone.0318686)
Supplement: S1 Document — (DOCX) [file pone.0318686.s001.docx]

|  | Call for projects  N°RCB: 2024-A00460-47  NCT : |
| --- | --- |

Digital mindfulness training program in smoking cessation and maintenance: a mixed-method pilot study

Study name: HowToMind

Version 2 of

Approved on 18/07/2024 by the CPP Ile de France I

Version 1.1 of

Approved on 19/04/2024 by the CPP Ile de France I

Search category :

Category 2: Interventional research with minimal risks and constraints

| PROMOTEUR  CHU Dijon Bourgogne  14 rue Gaffarel  21079 DIJON | PRINCIPAL INVESTIGATOR / COORDINATOR  Dr Anastasia DEMINA  University Hospital Addictology Department  CHU Dijon Bourgogne |
| --- | --- |

Protocol modification tracking table

| N°MSA | Protocol version | Modifications | CPP approval date | ANSM approval date |
| --- | --- | --- | --- | --- |
|  | Version 1 of 01/31/2024 | Initial version submitted to CPP |  | / |
|  | Version 1.1 of 03/29/2024 | Version 1 modified to comply with CPP | 19/04/2024 | / |
| MS 1 | Version 2 of 07/06/2024 | Modification of a non-inclusion criterion | 18/07/2024 | / |
|  |  |  |  |  |

SYNOPSIS

| Study title | Digital mindfulness training program in smoking cessation and maintenance: a mixed-method pilot study |
| --- | --- |
| Registration no. | 2024-A00460-47 |
| Developer | Dijon Bourgogne University Hospital |
| Investigators | Dr Anastasia DEMINA |
| Rational | Scientific background  Several approaches are effective in smoking cessation. Despite this, relapses are frequent and the proportion of people who remain abstinent is low (around 15-25% after 6 months' withdrawal). New tools to facilitate smoking cessation are therefore needed.  Mindfulness-based interventions (MBI) are effective in depression, stress reduction and chronic pain (Goldberg et al., 2017, Khoury et al., 2015, Hilton et al., 2017). There is also evidence of efficacy in smoking cessation with a relative risk at 1.88 95% CI [1.04; 3.40] of abstinence at 17 to 24 weeks after MBI (Oikonomou, 2016).  MBI usually takes the form of an 8-week program with weekly group sessions. However, only a handful of patients have sufficient availability to attend the full program. These programs are only accessible to a small number of patients due to the lack of trained professionals and non-reimbursed costs.  In response, smartphone applications have been developed and evaluated, but none has been shown to be effective in smoking cessation. What's more, none of the apps evaluated offered the equivalent of a classic MBI in 8 weeks.  Furthermore, it has now been demonstrated in the international literature that involving patients, users and relatives (PUPs) in the development and conduct of health research projects has become a means of achieving effective, high-quality integration of healthcare (Fusco, 2020) as well as improving the overall quality of health research (Shen, 2017). When PUPs are involved not as research subjects but as research partners in the health research process, this can lead to "significant changes in outcomes for patients and health systems, and the realignment of research processes and outcomes to be patient-centered" (Bird, 2020).  The involvement of PUPs has become a requirement for many funding programs and reviews, as well as an international health policy priority.  As a result, we are developing an eMind application - the first electronic MBI for smoking cessation that enables patients to follow a complete 8-week program and recreates as closely as possible the conditions of a classic MBI. The application will be available on the EXOLIS platform. This platform was proposed to us by the Agence Régionale de Santé Bourgogne - Franche Comté in partnership with GRADeS (Groupement Régional d'Appui au Développement de la eSanté).  The eMind application will be co-constructed: it will be based on a partnership research approach patient participation. A first version of the content has been created in the university hospital addictology department by healthcare professionals trained in mindfulness. This first version of the application - a beta version - will be modified according to patient feedback. To reward their contribution to the final version of the application, patients will be compensated in the form of a gift card. A session to present the results of the study and the new version of eMind will be organized once the modifications have been taken into account.  Research hypothesis  In this context, we formulate the hypothesis that, for our application to be optimally effective, taking into account the experience and expectations of the people concerned is one of the keys to better adherence to the application. |
| Experimental design | We propose a single-center mixed pilot study of the usability and adherence to the eMind application - beta version - in a population of smokers wishing to stop smoking.  Participants will have access to the eMind application to follow the 8-week MBI smoking cessation program. At the same time, they will receive a standard treatment combining nicotine patches and lozenges. |
| Objectives | - Main objective:   Evaluate the acceptability and usability of the eMind application, at 8 weeks, among smokers wishing to stop smoking. In addition, a qualitative component (focus groups) is planned to gather their feedback, their expectations and the determinants of the application's acceptability.   - Secondary objectives:   Study changes in tobacco consumption between inclusion and last follow-up (6 months).  Study the impact of eMind on smoking at 3 and 6 months after inclusion  Study the evolution of craving between inclusion and the last visit (12 weeks).  To evaluate the tolerance of smoking cessation accompanied by the eMind application .  Study the evolution of mindfulness practice at 3 and 6 months. |
| Study evaluation criteria | - Main evaluation criteria   The acceptability of the beta version eMind will be assessed by the regularity of its use. Based on the rhythm of the videos and audio provided by the application, we will define people who use eMind at least 4 times a week for 8 weeks as active users, those who use the application 1 to 3 times a week as occasional users, and those who no longer use the application after 8 weeks as users who have given up using the application.  Usability will be assessed using the MARS-F usability scale (Mobile App Rating Scale, French version) after 8 weeks' use of the application. The MARS-F usability scale is made up of 23 items rated from 1 to 5, and explores four objective domains (engagement, functionality, aesthetics, information quality) leading to a score between 0 and 95, and a 20-point subjective domain. Usability is considered good if at least 75% of users give a MARS-F score of at least 85 out of 115.  After 8 weeks of use, focus groups will be set up with active users, occasional users and those who have given up using the application, to gather their perceptions and experiences, with the aim of collectively establishing recommendations for the evolution of the beta version of our application.   - Secondary endpoints   The evolution of tobacco consumption between inclusion and 4 weeks, 8 weeks and 12 weeks will be measured using an electronic consumption calendar implemented in our application. A CO test will be performed at inclusion, 4 weeks, 8 weeks and 12 weeks. Smoking abstinence will be defined as the absence of tobacco consumption for 7 days prior to the assessment visit.  Assessment of tobacco consumption at 6 months, during a telephone call.  Craving intensity will be measured at baseline, 4 weeks, 8 weeks and 12 weeks using a visual analog scale (VAS) implemented in the application.  The evolution of mindfulness practice between inclusion and 4 weeks, 8 weeks, 12 weeks will be measured using an electronic consumption calendar implemented in our application.  Assessment of mindfulness practice at 6 months, during a telephone call.  Symptoms related to smoking cessation (cravings, difficulty concentrating, headaches, irritability, fatigue, hunger, constipation), use of nicotine replacement therapy (skin irritation, abdominal pain, hiccups, gastroesophageal reflux, sleep disorders, nausea, diarrhea, dizziness) or MBI will be collected throughout the study. |
| Expected number of participants | 60 participants are expected to take part  For the focus groups, 3 groups of 6 to 8 participants will be selected from the 60 participants in the study, based on a maximum variation sampling (age, gender, CSP) for each group. |
| Subject selection | - Inclusion criteria - Oral consent - Adults aged 18 and over - Smoker with a DSM-5 smoking disorder - With motivation to quit smoking - With daily access to a smartphone - Able to understand spoken and written French - Non-inclusion criteria - Person under legal protection (curatorship, guardianship) - Person subject to a safeguard of justice measure - People with cognitive disorders that prevent training - Currently using other smoking cessation treatments (burpropion, varenicline), apart from NRT - Suffering from an acute psychiatric or somatic disorder requiring hospitalization / not stabilized - With a contraindication to replacement therapy - Have an alcohol use disorder or consume illicit products   - Pregnant, parturient, breastfeeding or planning a pregnancy within the next 6 months next few months   - Person not affiliated to the national health insurance scheme |
| Assessment of expected benefits and risks | On an individual level, the practice of mindfulness, through learning the key messages of the 8-week MBI program, could lead to better tolerance of craving and a lower risk of relapse after smoking cessation.  On a collective level, the practice of mindfulness using eMind could be an important addition to standard care, extending access to mindfulness to as many patients as possible.  MBIs have been extensively studied in various clinical trials in the fields of mental health, addiction, pain and stress management. Literature data suggest that MBIs are well tolerated, provided that contraindications (any acute psychic or somatic condition) are respected.  Foreseeable side effects include those associated with smoking cessation (cravings, difficulty concentrating, headaches, irritability, fatigue, hunger, constipation) and the use of nicotine replacement therapy (skin irritation, abdominal pain, hiccups, gastroesophageal reflux, sleep disorders, nausea, diarrhea, dizziness). |
| Course of the study | - Chronological description of the study  1. Screening visit (university hospital addictology department at CHU Dijon Bourgogne)   - Informing the person wishing to participate in the research. Oral, free and informed consent  - Checking inclusion and non-inclusion criteria   1. Visit 1 (Inclusion, University Hospital Addictology Department, CHU Dijon Bourgogne). Inclusion visits will be grouped into 3 waves of 20 patients to enable focus groups to be set up and conducted from week 9 onwards.   - Checking inclusion and non-inclusion criteria  - Oral, free and informed consent collected by visit 1 at the latest.  - Collection of tobacco consumption over the last 4 weeks, measurement of CO  - Assessment of craving using VAS  - Presentation of the application, explanation of how it works and recommendations for use, decision on associated nicotine replacement therapy.  - After the visit, introduction to Mindfulness practice using the eMind application.   1. Visit 2 (4 weeks from inclusion, university hospital addictology department at CHU Dijon Bourgogne   - Collection consumption schedule, measurement of CO  - Assessment of craving using VAS  - Assessment of withdrawal symptoms and NRT use.  - Gather initial feedback on regular use of the application to plan focus groups   1. Visit 3 (8 weeks from inclusion, University Hospital Addictology Department, CHU Dijon Bourgogne   consumption schedules, measuring CO  - Assessment of craving using VAS  - Assessment of withdrawal symptoms and NRT use - Assessment of eMind usability using the MARS scale  - Gathering feedback on regular use of the application  The focus groups will be carried out between visit 3 and visit 4, to avoid any memory bias that might arise if they are carried out at too great a distance from use   1. Visit 4 (12 weeks from inclusion, University Hospital Addictology Department, CHU Dijon Bourgogne   - Collection consumption schedule, measurement of CO  - Assessment of craving using VAS  - Assessment of adverse effects   1. Telephone call 6 months after inclusion   - Tobacco consumption at 6 months  - A compendium of mindfulness practice |
| Statistical and qualitative analysis | - Justification of the number of people required   We plan to include 60 patients (N = 60). The number is based on the numbers commonly used in quantitative pilot studies. For the qualitative explorations, we estimate that the participation of 18 to 24 users will enable us to reach the data saturation threshold in the various focus groups. Given the potential for some participants to be lost to follow-up and to be unavailable to participate in focus groups, N = 60 seems to us to be the right number for the chosen methodology.   - **Tests used**   The MARS-F usability score will be calculated for each participant after the 8-week period of use. The score will be presented as a mean and standard deviation, accompanied by a 95% confidence interval.  The percentage of active, one-time and discontinued users will also be calculated, along with a 95% confidence interval calculated using the exact binomial method.  No test will be performed, but the confidence interval will indicate the degree of precision around the percentage and the mean  Secondary analyses will be descriptive and/or exploratory. Quantitative variables will be described in terms of means +/- standard deviations, medians and interquartile ranges. SAS v9.4 software will be used.  The focus groups will be recorded and transcribed in full. The corpus of qualitative data thus gathered will then be processed by thematic analysis by the sociologist in charge of the qualitative section. Multidisciplinary (addictologist, public health researcher, epidemiologist) coding triangulation sessions will take place when the descriptive analysis framework is put together, and ultimately when the thematic tree is created (axial coding). |
| Expected results and alternatives | We expect good acceptability of the application with its use throughout the treatment period.  Knowledge of the acceptability and usability of the beta version of eMind, as well as a study of the motivations and obstacles to its use, will provide us with information on the needs of the participants in terms of the operation of the eMind application. This will make it easier to overcome any obstacles to optimal involvement. These evaluations will enable us to make the necessary modifications before rolling out the program on a larger scale  Data on the effect size of our intervention will be used to calibrate a large-scale national intervention trial aimed at evaluating the effectiveness of the improved version of eMind, thanks to feedback from participants in the HowToMind study, using the participatory research approach. |
| Timetable | - duration of inclusion period: 21 months - total duration of participation for a person taking part in the research: 6 months  - total study duration (inclusion + possible follow-up): 27 months  Period of exclusivity for the person: no yes  Period of exclusion for the person:  no yes, duration: days |

STUDY DIAGRAM

Telephone follow-up

Consumption

Mindfulness practice

Autonomy phase, Practical application

Learning phase, Accompanied practice

Screening visit

Eligibility criteria verified,

Information

Inclusion visit 1

Obtaining informed consent,

Smoking,

Craving

Visit 2

Calendar of consumption, Craving,

CO measurement,

AR collection

Visit 3

Usability (MARS-F scale)

Connection data collection

Consumption calendar

Craving,

CO measurement,

EI collection

Visit 4

Consumption calendar

Craving

CO measurement

EI collection

Focus group

M 6

S1

S2

S3

S4

S5

S6

S7

S8

S9

S10

S11

S12

S13 S133

TABLE OF CONTENTS

Investigators and administrative structure 2

SYNOPSIS 4

STUDY DIAGRAM 10

1. STATE OF THE ART AND RATIONALE FOR THE STUDY 14

1.1. Current scientific knowledge and unanswered questions 14

**1.1.1.** **Smoking disorder** 14

**1.1.2.** **Reference treatments and new interventions :** 14

**1.1.3.** **Participatory healthcare** 16

**1.1.4. The eMind application** 16

1.2. Research hypothesis 17

1.3. Project originality and expected results 17

2. OBJECTIVES 18

2.1. Primary objective 18

2.2. Secondary objectives 18

3. JUDGING CRITERIA or INTEREST VARIABLES 18

3.1. Primary endpoint 18

3.2. Secondary endpoints 19

4. METHODOLOGY 19

4.1. Study diagram 19

4.2. Calculating the number of subjects required 19

**4.2.1.** **Justification** 19

**4.2.2.** **Feasibility** 20

4.3. Study population 20

**4.3.1.** **Inclusion criteria** 20

**4.3.2.** **Non-inclusion criteria** 20

4.4. Project feasibility 20

5. INTERVENTION 21

5.1. Intervention under study 21

5.2. Associated treatments 22

6. COURSE OF THE STUDY 22

6.1. Recruitment procedures 22

6.2. Information and free and informed consent, screening procedures 22

6.3. Inclusion criteria 22

6.4. Follow-up procedures / questionnaires 23

6.5. End of study 24

6.6. Feedback session 24

6.7. Technical description of measured parameters 24

6.8. Benefit-risk balance 24

6.9. Description of the rules for terminating a person's participation in research 25

6.10. Exclusivity - exclusion period 25

6.11. Study schedule 25

**6.11.1.** **Study duration** 25

**6.11.2.** **Study organization chart** 25

7. DATA MANAGEMENT 26

7.1. Privacy 26

7.2. Clinical data 26

7.3. Data collection 26

7.4. Quality and Safety Manager 26

7.5. Storage and archiving 26

8. STUDY COORDINATION 27

8.1. Steering Committee 27

8.2. Independent Supervisory Board 27

9. SAFETY ASSESSMENT 27

10. STATISTICAL ANALYSIS 27

10.1. Analysis strategy 27

10.2. Choice of analysis software 27

10.3. Significance threshold 27

10.4. Person responsible for analysis and place of analysis 27

11. ETHICAL and REGULATORY ASPECT 28

11.1. Ethical conduct of the study 28

11.2. Investigator's responsibilities 28

11.3. The promoter's responsibilities 28

**11.3.1.** **Comité de Protection des Personnes (CPP) and Competent Authority** 29

**11.3.2.** **Personal data protection** 29

**11.3.2.1.** **CNIL** 29

**11.3.2.2.** **Privacy** 29

**11.3.3.** **Insurance/Patient compensation** 29

**11.3.4.** **Authorization, persons in charge and study locations** 30

**11.3.5.** **Management information** 30

**11.3.6.** **Informing subjects about overall research results** 30

**11.3.7.** **Audit and inspection** 30

**11.3.8.** **Archiving** 30

12. STUDY FUNDING 30

12.1. Protocol financing 30

13. REPORTS AND PUBLICATIONS 30

13.1. Final report 30

13.2. Publication rules 31

14. BIBLIOGRAPHY 31

LIST OF ABBREVIATIONS AND DEFINITION OF TERMS

| Abbreviations | Meanings |
| --- | --- |
| AC | Competent authority |
| PPC | Comité de Protection des Personnes |
| EI | Undesirable effects |
| EIG | Serious adverse effect/event |
| CO | Carbon monoxide |
| CRF | Case Report Form |
| CTCAE | Common Terminology Criteria for Adverse Event |
| DSM-5 | Diagnostic and Statistical Manual of mental disorders, 5^th^ edition |
| EVA | Visual Analog Scale |
| HDRS | Hamilton Depression Rating Scale |
| HDS | Health Data Host |
| MARS-F | Mobile App Rating Scale, French version |
| MBSR | Mindfulness Based Stress Reduction |
| PUP | Patients, Users and Relatives |
| CBT | Cognitive and Behavioral Therapies |
| TLFB | Timeline Followback |
| TSN | Nicotine replacement therapy |
|  |  |
|  |  |
|  |  |
|  |  |
|  |  |
|  |  |
|  |  |
|  |  |
|  |  |
|  |  |

# STATE OF THE ART AND RATIONALE FOR THE STUDY

## Current scientific knowledge and unanswered questions

### **Smoking disorder**

Tobacco use disorder is defined by the DSM-5 through eleven diagnostic criteria (the presence of two criteria is sufficient to establish the diagnosis). These criteria group together elements suggestive of loss of control over tobacco consumptionas well as the development of tolerance and the appearance of withdrawal signs on stopping consumption. This latest version of the DSM, adding the new criterion of craving (defining irrepressible urges to consume), establishes the central place of this painful symptom in addictive disorders.

Smoking is one of the world's leading causes of preventable illness and death (1). Directly responsible for 75,000 deaths a year in France, it is the leading cause of preventable death in our country (2). In Europe, tobacco is responsible for 25-30% of cancer deaths (3). Worldwide, 5 million deaths are caused by tobacco. Public health estimates suggest that if current smoking levels continue, 1 billion deaths will be attributable to tobacco during the 21st century. The costs generated by smoking are colossal (4).

Of all tobacco users, around half suffer from a smoking disorder. Smoking disorders are characterized by neurobiological changes in motivation and learning, making it extremely difficult to stop smoking.

### **Reference treatments and new procedures:**

Recently, several therapeutic approaches have demonstrated a significant impact on smoking cessation and maintenance of abstinence (nicotine replacement therapy (NRT), Varenicline, Buproprion).

NRT remains the first-line treatment in France. This standard treatment combines a transdermal form (patch) for continuous delivery of nicotine over 24 or 16 hours, with an oral form (lozenges, gum, spray, etc.) for delivery of nicotine at times when cravings are felt. The dosage of NRT is adjusted according to cravings, with the right dose ensuring that cravings disappear. Withdrawal signs and cravings are thus well controlled, while nicotine reinforcement is reduced.

While NRT remains the reference treatment, there are also so-called addictolytic treatments. Varenicline is a partial nicotinic receptor agonist, with the mechanism of action of reduced craving and withdrawal signs associated with reduced positive reinforcement linked to tobacco use. Another treatment, Bupropion, an antidepressant, reduces craving and withdrawal signs through its action on dopamine, norepinephrine and nicotine/acetylcholine receptors.

Despite these medicinal treatment options, relapse is frequent, and only 15-25% of smokers who have quit remain abstinent after 6 months (5). New treatments, including those adjuvant to standard therapies, are therefore essential.

In 1979, Jon Kabat-Zinn created a Mindfulness training program called Mindfulness Based Stress Reduction (MBSR). Mindfulness is understood as an altered state of consciousness enabling the direct experience of the present moment through focused attention to internal and external stimuli. The neurobehavioral effects of Mindfulness training are mediated by attentional allocation mechanisms, translating into efficacy indicators in depression, stress reduction and chronic pain management (6,7).

Several Mindfulness-Based Interventions (MBIs) currently exist, such as MBSR, Mindfulness-Based Cognitive Therapy (MBCT) and Mindfulness-Based Enhanced Recovery (MORE), to name but a few. MBIs usually involve weekly group sessions lasting several weeks (usually eight weeks), combined with independent training between sessions.

Neurobiological research highlights the failure of prefrontal control in addictive processes: the reduction of prefrontal control by nicotine leads to compulsive smoking. Mindfulness training aims to bring attention back to the present moment, reinforcing decision-making mechanisms not by impulsivity, but with full awareness, thereby combating the automatic behavior that is addictive behavior. MBIs also aim to facilitate the acceptance and tolerance of discomfort, which could reduce craving and thus diminish the need to consume to alleviate that craving.

MBIs have been studied in numerous clinical trials. A recent meta-analysis established a small but significant effect of mindfulness-based relapse prevention, a program specifically addressing addiction issues, on withdrawal symptoms with an effect size of -0.13 (95% CI -0.19 to -0.08, I2 = 0%) (13). MBIs have an effect on smoking cessation with an estimated RR of 1.88 at 95% [1.04; 3.40] for 7-day punctual abstinence 17-24 weeks post-intervention (11).

The preliminary efficacy of MBIs suggests the transdiagnostic quality of these interventions. MBIs appear to have a significant effect on craving and may influence a variety of other potential therapeutic targets of interest in addictology, such as stress, cue reactivity, attentional biases and psychological flexibility. Finally, these interventions are highly acceptable to patients, with no significant adverse effects. Today, MBIs count as evidence-based interventions and are included in the classification of so-called third-wave cognitive-behavioral therapies (CBTs).

Conventional MBIs show promising results for smoking cessation, but they also require the presence of trained professionals and available rooms. What's more, these programs may be unfeasible for many patients: only a handful of patients have sufficient availability to attend the full program. In addition, conventional MBIs are not accessible to many patients due to the non-reimbursed costs of these procedures.

To overcome these practical problems of accessibility to conventional MBIs, several smartphone applications have been developed. So far, none of them has demonstrated significant benefits for smoking cessation, unlike conventional MBIs.

In the literature, the study by Hoover et al (20), compared a face-to-face or digital Mindfulness-based intervention (during the Covid epidemic) with the standard intervention in healthcare students at risk of burnout. Both Mindfulness-based interventions were effective in reducing stress perception. In the protocol of this study, there was no randomization between conventional and digital Mindfulness, the digital intervention having been added in the manner required by COVID19.

The article by Mrazek et al, 2019 (19) takes stock of the benefits and "challenges" associated with digital Mindfulness, highlighting the accessibility of digital interventions with, in particular, flexibility in choosing a training schedule. Several qualitative studies support the advantage of temporal flexibility in connection with digital versus conventional interventions, asking participants to free up several hours a week for several weeks (20, 21)

Recently, Judson Brewer and his team at Yale University developed a smartphone app called "Craving to Quit" using Mindfulness for smoking cessation (8). His team conducted a study suggesting that the association between craving and smoking was reduced with use of the app (9). However, "Craving to Quit" only offers a partial three-week program, and none of the existing apps offers an equivalent for a classic 8-week program.

### **Participatory healthcare**

It has now been demonstrated in the international literature that involving patients, users and relatives (PUPs) in the development and conduct of health research projects has become a means of achieving effective, high-quality healthcare integration (Fusco, 2020) as well as improving the overall quality of health research (Shen, 2017). When PUPs are involved not as research subjects but as research partners in the health research process, this can lead to "significant changes in outcomes for patients and health systems, and the realignment of research processes and outcomes to be patient-centered" (Bird, 2020).

The involvement of PUPs has become a requirement for many funding programs and journals, as well as a health policy priority at international level. In the international literature on e-health devices aimed at facilitating smoking cessation, participatory studies are essential steps prior to the implementation of large-scale efficacy studies (Asfar, 2021; Oakley-Girvan, 2022)

### **1.1.4. The eMind application**

Professionals from the addictology department at the CHU Dijon Bourgogne, trained in both addictology and Mindfulness, have created Mindfulness content that has been implemented on a digital platform in the form of an e-health application, called eMind. This implementation was made possible thanks to collaboration with public players such as the Agence Régionale de la Santé Bourgogne Franche Comté (ARS BFC) and the Groupement régional d'aide au développement de la e-santé (GRADeS). The latter are certified "Hébergeur de Données de Santé" (HDS) for all the following activities:

- The provision and maintenance in operational condition of physical sites to host the hardware infrastructure of the information system used to process health data;
- The provision and maintenance in operational condition of the hardware infrastructure of the information system used to process health data;
- Provision and maintenance in operational condition of the information system's application hosting platform;
- Provision and maintenance in operational condition of the virtual infrastructure of the information system used to process health data;
- Administration and operation of the information system containing health data;
- Health data backup.

The eMIND application includes weekly educational modules in the form of videos and audio exercises for daily practice. The application is intended for clinical use as a complement to standard treatment. As such, quality requirements such as data security, the scientific value of the information delivered, as well as ease of use or usability must apply.

The content of our application has been created by trained healthcare professionals, guaranteeing the quality of the information delivered. With regard to usability or ease of use, the French health authority (Haute Autorité de Santé) recommends that it be evaluated with the target population (10). A high level of usability is expected.

We propose to evaluate our original digital MBI by measuring the acceptability and usability of this e-health tool in a population of smokers undergoing smoking cessation with standard treatment. We will combine this with a partnership research approach involving patient participation, in order to co-construct the final version of the application.

## Research hypothesis

In this context, we formulate the hypothesis that, for our application to be optimally effective, taking into account the experience and expectations of the people concerned is one of the keys to better adherence to the application.

## Project originality and expected results

We propose a single-center, mixed-methods pilot study to assess the acceptability and usability of the eMind application in a population of smokers wishing to quit.

eMind is the first Mindfulness application for smoking cessation to enable patients to follow a complete 8-week MBI that recreates as closely as possible the conditions of a classic MBI. It differs from other Mindfulness applications in that most existing applications offer only elements of Mindfulness programs, without the rich, intensive and progressive training process of a standard 8-week program. Compared with a traditional face-to-face program, Mindfulness practice via eMind is said to encourage patients' autonomy in deciding when to practice, and to be more compatible with their life rhythms.

The eMind application will be co-constructed: it will be based on a partnership research approach with patient participation. A first version of the content has been created in the university hospital addictology department by healthcare professionals trained in mindfulness. This first version of the application - a beta version - will be modified according to patient feedback. To reward their contribution to the final version of the application, patients will be compensated in the form of a gift card. A session to present the results of the study and the new version of eMind will be organized once the modifications have been taken into account.

Knowledge of the acceptability and usability of the beta version of eMind, as well as a study of the motivations and obstacles to its use, will provide us with information on the needs of the participants in terms of the operation of the eMind application. This will make it easier to overcome any obstacles encountered, so as to achieve optimum involvement. These evaluations will enable us to make the necessary adjustments before rolling out the program on a larger scale.

Data on the effect size of our intervention will be used to calibrate a large-scale national intervention trial aimed at evaluating the effectiveness of the improved version of eMind, thanks to feedback from participants in the HowToMind study, as part of the participatory research approach.

Our application could offer new therapeutic perspectives for smokers. This treatment could benefit the 12.2 million smokers in France (1 billion smokers worldwide). It is therefore crucial to guarantee the usability of our application, so that we can later explore its efficacy in a large multicenter randomized controlled trial

In terms of public health gains, even a small effect would be significant given the prevalence of smokers in the French population. For example, if the minimum detectable effect were achieved for eMind, the 1-year abstinence rate after smoking cessation would rise from 16% to 25%. Given that almost 25% of smokers have tried to quit in the past year, and that 27% of the population aged 18 to 75 currently smoke, this means that around 300,000 more smokers will maintain their abstinence each year if this application is widely available (17).

# OBJECTIVES

## Primary objective

Evaluate the acceptability and usability of the eMind application, at 8 weeks, among smokers wishing to stop smoking. In addition, a qualitative component (focus groups) is planned to gather their feedback, their expectations and the determinants of the application's acceptability.

## Secondary objectives

Study changes in tobacco consumption between inclusion and last follow-up (6 months).

Study the impact of eMind on smoking at 3 and 6 months after inclusion.

Study the evolution of craving between inclusion and the last visit (12 weeks).

Evaluate the tolerance of smoking cessation supported by the eMind application.

Study the evolution of mindfulness practice at 3 and 6 months.

# JUDGING CRITERIA or INTEREST VARIABLES

## Primary endpoint

The acceptability of the beta version of eMind will be assessed by the regularity of its use. We will define people who use eMind at least 4 times a week for 8 weeks as active users. Those using the application 1 to 3 times a week will be defined as occasional users, and those no longer using the application after 8 weeks will be defined as users who have given up using the application.

Usability will be assessed using the MARS-F usability scale (Mobile App Rating Scale, French version) after 8 weeks' use of the application. The MARS-F usability scale is made up of 23 items rated from 1 to 5, and explores four objective domains (engagement, functionality, aesthetics, information quality) leading to a score ranging from 0 to 95, and a subjective domain with 20 points. Usability is considered good if at least 75% of users award a MARS-F score of at least 85 out of 115.

After 8 weeks of use, focus groups will be set up with active users, occasional users and those who have given up using the application, to gather their perceptions and experiences with a view to collectively establishing recommendations for the evolution of the beta version of our application.

## Secondary endpoints s

The evolution of tobacco consumption between inclusion and 4 weeks, 8 weeks and 12 weeks will be measured using an electronic consumption calendar implemented in our application. A CO test will be performed at inclusion, 4 weeks, 8 weeks and 12 weeks. Smoking abstinence will be defined as the absence of tobacco consumption for 7 days prior to the assessment visit. A recent consensus of experts has recommended the use of 7-day punctual abstinence as an endpoint, as it is less affected by recall bias and missing data than continuous abstinence (16).

Assessment of tobacco consumption at 6 months, during a telephone call.

Craving intensity will be measured at baseline, 4 weeks, 8 weeks and 12 weeks using a visual analog scale (VAS) implemented in the application.

The evolution of mindfulness practice between inclusion and 4 weeks, 8 weeks, 12 weeks will be measured using an electronic consumption calendar implemented in our application.

Evaluation of mindfulness practice at 6 months, during a telephone call.

Symptoms related to smoking cessation (cravings, difficulty concentrating, headaches, irritability, fatigue, hunger, constipation), use of nicotine replacement therapy (skin irritation, abdominal pain, hiccups, gastroesophageal reflux, sleep disorders, nausea, diarrhea, dizziness) or MBI will be collected throughout the study.

# METHODOLOGY

## Study diagram

We propose a single-center pilot study to characterize the acceptability and usability of the beta version of the eMind application among smokers. The effect size of this intervention on the maintenance of smoking abstinence will be collected as a secondary endpoint.

## Calculating the number of subjects required

### **Justification**

We will include 60 patients (N = 60). The number is based on the sample size commonly used in pilot studies. In addition, we are planning three different focus groups with 6 to 8 participants in each. Given the potential for some participants to be lost to follow-up and unavailable to participate in focus groups, N = 60 seems to us to fit in well with the chosen methodology

### **Feasibility**

The project leader is an addictology doctor trained in Mindfulness and practicing in an addictology department with extensive experience in clinical research in tobacco (recruitment of 117 participants over 24 months for the Tabacstim 2 and 3 projects).

## Study population

### **Inclusion criteria**

- Person having given oral consent

- Adults aged 18 and over

- Smoker with a DSM-5 smoking disorder

- With motivation to quit smoking

- With daily access to a smartphone

- Able to understand written and spoken French

### **Non-inclusion criteria**

- Person under legal protection (curatorship, guardianship)

- Person subject to a safeguard of justice measure

- People with cognitive disorders that prevent training.

- Currently using other smoking cessation treatments (burpropion, varenicline), apart from NRT.

- Suffering from an acute psychiatric or somatic disorder requiring hospitalization / not stabilized

- With a contraindication to replacement therapy

- Have an alcohol use disorder or consume illicit products

- Pregnant, parturient, breastfeeding or planning to become pregnant within the next 6 months

- Person not affiliated to the national health insurance scheme

## Project feasibility

We expect a high level of feasibility for our intervention, as it will be of light intensity, favoring participant compliance. In addition, a large number of smokers in the general population will enable our recruitment. In our trial, participants will be recruited by healthcare professionals, which favours the suitability of the intervention for each participant.

This research is being carried out as part of the activities of the Addictology Department at Dijon University Hospital. This department is a level 3 facility, which positions the hospital as a regional referral center in the field of addictions. It offers a comprehensive range of care services, combined with the research and teaching missions of a hospital such as Dijon Bourgogne University Hospital. The Addictology Department at Dijon University Hospital has a specialized tobacco consultation. This is by addictology physicians and nurses qualified in the field. An active file of smokers intending to stop smoking is clearly identified among the patients consulting the service (smoking = 30% of consultants). In addition, the department has extensive experience in clinical studies on tobacco.

The inclusion period will last 21 months. Follow-up will last 6 months. The study will therefore last 27 months.

# INTERVENTION

## Study intervention

The study consists of the following two phases:

1. Supported learning phase (8 weeks) :

This phase will consist of Mindfulness training via the beta version of the eMind smartphone application, in addition to the standard treatment (TSN). The eMind application contains 8 thematic modules linked to both Mindfulness training and tobacco consumption (one module per week). Tools for daily practice will be available in audio/video and text formats (PDF documents). For each module, key messages will be delivered through a weekly video, and Mindfulness sessions ranging from 5 to 15 minutes each, available for daily practice (body scan, movement, breathing, etc.). The modules will target specific elements of addiction such as craving and automatic behavior, as well as transdiagnostic elements such as emotion, self-judgment and acceptance.

During this phase, each week a new module will be made available to the participant. Previously delivered modules will remain available for consultation at the patient's convenience.

*Summary of topics covered by the 8 learning modules :*

- Week 1: Introduction to Mindfulness
- Week 2: Addiction and automatic behavior
- Week 3: Craving
- Week 4: Emotion
- Week 5: Shame and judgment
- Week 6: Acceptance
- Week 7: Relapse
- Week 8: Building your practice

1. Independent practice phase :

The eMind application is designed to help participants acquire the skills they need to practice Mindfulness independently. Therefore, after the 8-week learning period, no new modules will be made available to participants. However, participants will be able to access all eMind resources as part of their autonomous practice

The smoking schedule (0 = no smoking, 1 = 1 cigarette, etc.; daily fill-in) and a craving assessment tool (VAS between 0 and 10; weekly fill-in) will also be implemented in the application. Similarly, the mindfulness practice calendar will be collected via the app (Do you practice mindfulness today: YES/NO, daily fill-in). In the event of non-completion of the calendar or the craving VAS, the principal investigator will receive alerts and will be able to contact the participant to offer help in the event of difficulties.

Through the eMIND application, two other types of alert will be set up:

- Patient reminders if video content is not viewed
- Alerts on the clinician interface in the event of non-viewing of video content by the patient.

## Associated treatments

Participants in this study will receive NRT in transdermal (patch) and oral (lozenge) forms, which is a standard treatment in this indication. The evolution of NRT doses will depend on the patient's clinical assessment, thus reproducing real NRT treatment conditions. The nicotine patch is applied to dry skin every 24 hours. Lozenges will be used as needed, up to 10 lozenges per day.

# COURSE OF THE STUDY

## Recruitment procedures

Eligible patients are identified prospectively by an investigating physician. These are generally patients seen as part of a psychiatric or addictology consultation at their own request, or referred by a general practitioner, or as part of a psychiatric or addictology liaison at the CHU

## Information and free and informed consent , screening methods

If the patient meets the eligibility criteria, the investigating physician will present the study during a tobaccology consultation in the Addictology Department of the CHU Dijon Bourgogne. He will inform the patient of the nature of the research, its objectives, methodology, duration, expected benefits, constraints and foreseeable risks, in accordance with article L1122-1 of the CSP. The investigating physician will give the patient the information note. Patients will be free to ask any questions they may have about the research, will be allowed a period of reflection and will be informed of their right to refuse to take part in the research or to withdraw their consent at any time without prejudice and without having to justify the reasons for their decision.

If the patient agrees, oral, free and informed consent will be obtained from one of the physician-investigators. The participant will be free to ask any questions about the research, and will be given a period of time reflect on his or her decision. If the patient agrees, his or her oral, free and informed consent will be obtained by one of the physician-investigators. A copy of the information note will be given to the patient, and two copies of the attestation d'obtention de consentement oral (ACOP) will be completed by the physician-investigator and kept by the patient and the investigator respectively. The investigator must record in each patient's medical file the fact that the patient has been informed and is included in a research protocol.

## Inclusion criteria

During the inclusion visit (Visit 1), the inclusion and non-inclusion criteria will be verified, oral informed consent will be obtained from the investigating physician, tobacco consumption over the last 28 days will be assessed, exhaled CO will be measured, and craving intensity will be assessed using a visual analog scale (VAS). During this visit, the eMind application will be presented and the participant will be given an access code to download it. The first connection to the application will be made with the investigator, in order to enter the inclusion number, which cannot be changed afterwards. Following this visit, participants will begin their MBI practice using the application associated with nicotine replacement therapy.

The investigator will collect socio-demographic information (age, sex, professions and socio-professional categories, urban/rural residence), medical and surgical history, usual treatments, as well as tobacco consultation data.

Inclusion visits will be grouped into 3 waves of 20 patients, to enable focus groups to be set up and conducted from week 9 onwards.

## Follow-up / questionnaires

Participants will receive follow-up visits at 4 and 8 weeks of inclusion, as follows:

- Visit 2 (4 weeks from inclusion, university hospital addictology department at CHU Dijon Bourgogne)

- Collection of consumption schedule, measurement of CO

- Assessment of craving using VAS

- Assessment of withdrawal symptoms and NRT use

- Gather initial feedback on the regularity of use of the application in order to plan the formation of focus groups.

- Visit 3 (8 weeks from inclusion, University Hospital Addictology Department, CHU Dijon Bourgogne)

- Collection of consumption schedule, measurement of CO

- Assessment of craving using VAS

- Assessment of withdrawal symptoms and NRT use

- Assessment of eMind usability using the MARS scale

- Collect feedback on the regularity of application use.

The focus groups will be carried out between visit 3 and visit 4, in order to avoid any memory bias that might arise if they are carried out too far after the product has been used.

In order to obtain an overall assessment of feedback, expectations and the determinants of the application's acceptability and impact on participants, the following topics will be addressed during the focus groups:

- Knowledge/representations of eHealth tools: history, practice in what context, general opinion of eHealth tools

- Practical application and access to the tool: access to the tool (connection, handling, navigation, etc.), general appearance, flexibility and adaptability of use, suitability of the tool to the smoking cessation context

- Perceived impact on smoking behavior: changes in smoking habits, obstacles, helping factors, shortcomings.

- Improvements to the tool and addictology follow-up: additions/improvements to the tool/follow-up visits, application reminders, online tools for self-monitoring of consumption, access to healthcare professionals.

Visit 4 to 12 weeks after inclusion (M3). During this visit, the following tests will be performed:

- Collection of consumption schedule, measurement of CO

- Assessment of craving using VAS

- Assessment of withdrawal symptoms and NRT use

## End of study

Telephone call 6 months after inclusion, during which the following information will be collected:

- Tobacco consumption at 6 months
- A compendium of mindfulness practice

## Feedback session

Once the results have been analyzed and the modifications made in line with the participants' feedback, we will propose a feedback session to present the results of the study and the new version of eMind.

##

## Technical description of measured parameters

- Tobacco consumption calendar, rated 0 = no consumption, 1 = 1 cigarette, etc., integrated into the eMind application: daily completion by the patient (with reminder notification).
- EVA Visual Analog Scale ("craving") rated from 1 to 10, integrated into the eMind application: weekly completion by the patient (with reminder notification).
- MARS-F Mobile Application Evaluation Scale composed of 23 items scored from 1 to 5 and exploring four objective domains (engagement, functionality, aesthetics, information quality) leading to a score between 0 and 95 and a 20-point subjective domain: collected at each visit by the investigator [15].
- Mindfulness practice calendar, integrated into the eMind application: daily completion by the patient (with reminder notification).

## Benefit-risk balance

On an individual level, the practice of mindfulness, through learning the key messages of the 8-week MBI program, could lead to better tolerance of craving and a lower risk of relapse after smoking cessation.

On a collective level, the practice of mindfulness using eMind could be an important addition to standard care, extending access to mindfulness to as many patients as possible.

MBIs have been extensively studied in various clinical trials in the fields of mental health, addiction, pain and stress management. Literature data suggest that MBIs are well tolerated, provided that contraindications (any acute psychic or somatic condition) are respected.

Foreseeable side effects include those associated with smoking cessation (cravings, difficulty concentrating, headaches, irritability, fatigue, hunger, constipation) and the use of nicotine replacement therapy (skin irritation, abdominal pain, hiccups, gastroesophageal reflux, sleep disorders, nausea, diarrhea, dizziness).

We expect the application to be well accepted and used throughout the treatment period. Knowledge of the acceptability and use of the eMind tool, as well as a study of the motivations and obstacles to its use, will help us to make the necessary adjustments before rolling out the program on a larger scale.

## Description of the rules for discontinuing a person's participation in research

All those included will be followed until the end of the study, except for patients who have withdrawn their consent.

## Exclusivity - exclusion period

| Exclusivity period  If yes, the patient cannot participate simultaneously in another research project | yes  no |
| --- | --- |
| Exclusion period  If yes, the patient may not participate in other research at the end of this one for a defined period of time. | yes  Duration: ....  no |
| Amount of compensation received by a patient for taking part in a focus group | 30€ |

## Study schedule

### **Study duration**

| Inclusion time | 21 months |
| --- | --- |
| Follow-up time for one person | 6 months |
| Total study duration  (from 1st inclusion to last patient follow-up) | 27 months |

### **Study organization chart**

|  | Screening | Visit 1  Inclusion | Visit 2  S4 | Visit 3  S8 | Visit 4  S12 | Phone call  M6  (End) |
| --- | --- | --- | --- | --- | --- | --- |
|  | J-x | J0 |  |  |  |  |
| Informed consent | X | X |  |  |  |  |
| Clinical examination | X | X |  |  |  |  |
| Patient questionnaires |  | X | X | X | X |  |
| CO test |  | X | X | X | X |  |
| Withdrawal-related symptoms |  |  | X | X | X |  |
| Dispensing treatment |  | X | X | X | X |  |
| Tobacco consumption |  | X | X | X | X | X |
| Mindfulness practice |  | X | X | X | X | X |

# DATA MANAGEMENT

## Privacy

Only the patient's code is entered in the CRF. This consists of the patient initials (first letter of the surname and first letter of the first name), the center number and the inclusion rank number. When extracting data, only the rank number is retained.

Ownership and use of the data will be exclusive to the Study Sponsor.

## Clinical data

The coding and correspondence of these variables will be available in a document from the database manager. This document will be drawn up before the database is built.

## Data collection

The data collected during the medical visits are those classically collected as part of smoking cessation management (age of smoking initiation, number of cigarettes consumed per day, history of smoking cessation with medicinal and non-medicinal means used, duration of longest abstinence, e-cigarette use, smoker or vapoteur entourage at the inclusion visit; CO measurement).The data will also be used to determine the regularity of use of the application (active user, occasional user, discontinued user). These data will be collated in an electronic CRF (e-CRF) created from CleanWeb software by a data manager. Data entry will be directly accessible online at the following address, after logging in with a login and password

<https://chu-dijon.tentelemed.com/Ctms-chud/portal/login>

Data concerning tobacco consumption and craving intensity (data commonly collected as part of standard care), as well as mindfulness practice, will be collected by participants on the application using the EXOLIS platform, which is certified to host health data. Regular use of the application will be recorded using the EXOLIS platform.

The qualitative data collected during the focus groups will be recorded on a digital dictaphone not connected to the Internet. They will then be transcribed verbatim, respecting the anonymity of the participants, and securely hosted on the servers of CHU Dijon Bourgogne until they are deleted once the results have been used.

## Quality and Safety Manager

Security, quality and access to study data will be managed by the USMR data manager.

Data entry will be validated live by dynamic consistency checks implemented during database construction.

A series of queries will be carried out periodically during the project by a data manager to validate the consistency of all data. These queries will be iteratively repeated until an error-free database is obtained.

## Storage and archiving

Data hosting via CleanWEB software is provided by Telemedicine Technologies via its secure Internet hosting platform.

A copy of the frozen database extraction file in "csv" format will also be stored on a Dijon Bourgogne CHU server, password-protected by the data manager.

# STUDY COORDINATION

## Steering Committee

Not applicable

## Independent Supervisory Board

Not applicable

# SAFETY ASSESSMENT

In the context of a category 2 clinical study, patient safety is monitored in accordance with the institution's rules of standard care and procedures.

# STATISTICAL ANALYSIS

## Analysis strategy

Analyses will be carried out on the frozen database resulting from the reconciliation of data collected on Cleanweb and Exolis.

Quantitative component

The MARS-F usability score will be calculated for each participant after the 8-week period of use. The score will be presented as a mean and standard deviation, accompanied by a 95% confidence interval.

The percentage of active, one-off and discontinued users will also be calculated, along with a 95% confidence interval calculated using the exact binomial method.

Secondary analyses will be descriptive and/or exploratory. Quantitative variables will be described in terms of means +/- standard deviations, medians and interquartile ranges.

Quality

The focus groups will be recorded and transcribed in full. The resulting corpus of qualitative data will then be processed by thematic analysis. Multidisciplinary (addictologist, public health researcher, epidemiologist) coding triangulation sessions will take place when the descriptive analysis framework is put together, and ultimately when the thematic tree (axial coding) is created.

## Choice of analysis software

Quantitative component: SAS software version 9.4

Qualitative component: n-Vivo software

## Significance threshold

Quantitative: No statistical tests will be carried out, but the confidence interval will indicate the degree of precision around the percentage and the mean.

## Person responsible for analysis and place of analysis

The analyses will be carried out by the DRCI Research Methodology Support Unit team at Dijon University Hospital, under the responsibility of Dr A Soudry-Faure for the quantitative aspect and Dr Meunier-Beillard for the qualitative aspect, in collaboration with Dr Demina, Addictology Department, Dijon Bourgogne University Hospital.

# ETHICAL AND REGULATORY ASPECT

## Ethical conduct of the study

The planning and conduct of this study are governed by French law (law no. 2012-300 of March 5, 2012 relating to research involving the human person, amended by order no. 2016-800 of June 16, 2016 and its implementing decrees). This research may only begin once all legal provisions relating to obligations prior to the implementation of research have been complied with. The study will be conducted in accordance with the ethical principles of the Declaration of Helsinki and the recommendations of Good Clinical Practice.

**In accordance with article L1121-1 of the French Public Health Code, this study constitutes** *category 2* **research involving the human body***,* **in that** it is interventional research involving only minimal risks and constraints, the list of which is set by order of the Minister of Health.

## Investigator's responsibilities

- The investigator undertakes that this study will be carried out in compliance with law no. 2012-300 of March 5, 2012 relating to research involving the human person as amended by order no. 2016-800 of June 16, 2016 and their implementing decrees, the Declaration of Helsinki, Good Clinical Practice. All data, documents and reports may be the subject of audits and regulatory inspections, without any objection to medical confidentiality.
- The investigator will inform the volunteers of the objectives and constraints of the study, and of their right to refuse to participate or to leave the study at any time. Once this information has been given to the subject, the investigator will ensure that he/she has fully understood the implications of participating in the study, his/her oral consent will be obtained by one of the investigators, and two copies of the Attestation d'obtention de consentement oral (ACOP) will be completed*.* One copy of the signed ACOP will be given to the subject, and the other original copy will be retained by the investigator.

The consent process will be documented in the medical record.

- All information collected is confidential and may not be divulged. The investigator will ensure that the anonymity of each volunteer participating in the study is guaranteed. No information allowing identification of individuals will be communicated to third parties other than those, representing the sponsor and the Ministry of Health, legally authorized to hold this information (and who are bound by professional secrecy).

## The promoter's responsibilities

CHU Dijon Bourgogne is promoting this study.

In accordance with applicable regulations, the developer undertakes to carry out all the operations required of it:

- **Registration of the study** with ANSM (n°ID-RCB)*.*
- **Information or request for authorization from the French National Agency for the Safety of Medicines and Health Products (ANSM)**
- **Submission to the Comité de Protection des Personnes**.
- **Declaration or request for authorization from the Commission Nationale Informatique et Liberté**.
- **Insurance coverage** for interventional research as defined in 1° and 2° of Article L1121-1 of the French Public Health Code and in European regulations EU 2017/745 and EU 536/2014
- **Substantial modification :**

After the study has begun, any substantial modification of the protocol initiated by the investigator must be submitted to the sponsor, who must obtain a favorable opinion from the CPP before implementing it.

- **Declaration of Serious Breaches on CTIS**
- **Declaration of study start defined by 1st inclusion**
- **Declaration of end of study defined by last patient follow-up**

### **Comité de Protection des Personnes (CPP) and Competent Authority**

The study cannot begin without the authorization of a Committee for the Protection of Individuals.

The protocol was approved by the CPP Ile de France I on 19/04/2024.

On receipt of the CPP's favorable opinion, the sponsor will inform the ANSM.

Authorization from the Comité de protection des personnes lapses if, within two years of the favorable opinion, the research has not begun (i.e. no person has been included in the protocol).

Neither the investigator nor the sponsor may modify this protocol without the prior written agreement of the other party. If substantial modifications are to be made, they must be set out in an amendment to the protocol.

This amendment will be applied once it has been approved by the PPC.

### **Personal data protection**

The computer file used to carry out this research will be subject to a commitment of compliance with the C.N.I.L. in application of the law "informatique et liberté", *law n°78-17 of January 6, 1978 relating to data processing, files and freedoms as amended and the General Regulation on the Protection of Personal Data (RGPD), adopted at European level, and entered into force on May 25, 2018*.

### **CNIL**

The processing of the information gathered during this study will be done in compliance with the MR001 reference methodology. Declaration n° 2210226 v 0 dated December 03, 2018.

### **Privacy**

In accordance with the provisions of article R5121-13 of the French Public Health Code, the investigator and any person called upon to collaborate in the studies are bound by professional secrecy, in particular with regard to the nature of the products studied, the studies, the persons involved and the results obtained, subject to the provisions of article L1123-14 of the French Public Health Code.

Without the agreement of the promoter (CHU Dijon Bourgogne), they may only provide information on the study to the Health Authorities, including inspectors as mentioned in article R5121-13 of the Public Health Code.

No oral or written comments will be made on the studies without joint authorization from the coordinating investigator and the sponsor (Dijon Bourgogne University Hospital).

Medical data concerning patients will only be transmitted to the promoter and, where applicable, to the relevant health authorities, under conditions guaranteeing confidentiality. Patients may exercise their right of access and rectification by contacting their investigator.

### **Insurance/Patient compensation**

The sponsor has taken out insurance to cover its civil liability in the event of any harmful consequences arising from this research.

In accordance with current legislation (article L. 1121-10 of the French Public Health Code), each patient is insured against any deterioration in his or her state of health that may result from participation in the study.

CHU Dijon Bourgogne's insurance company is Relyens Policy no. 129.234.

The investigator must immediately report to the Dijon Burgundy University Hospital's Clinical Research Department any complaint made by a patient that may be related to the study. The Director of Clinical Research will forward the complaint to the Legal Department.

### **Authorization, persons in charge and study locations**

An Authorization of Place is required for the research mentioned in 1° of article L. 1121-1 of the French Public Health Code, carried out outside the place of care or in hospital departments when this research requires procedures other than those they usually carry out as part of their activity, or when this research is carried out on people presenting a clinical condition distinct from that for which the department is responsible.

The head of research is the principal investigator at each center.

### **Management information**

The sponsor has ensured that the head of the trial center has been informed prior to the start of the study, and that an agreement has been drawn up with each health facility participating in the protocol.

### **Informing subjects about overall research results**

At the end of the study, if the volunteer so wishes, he or she may be informed of the overall results of the research (Article L1122-1, last paragraph). There is no possibility for people to be informed of the individual results of the research, but they can be informed of their medical data.

The patient must make the request in writing to the investigating physician.

### **Audit and inspection**

The investigators agree to comply with the requirements of the sponsor and the Competent Authority regarding an audit or inspection of the study.

The audit may be applied to all stages of the study, from protocol development to publication of results and classification of data used or produced as part of the study.

### **Archiving**

At the end of the study, all documents related to the study (including copies of observation notebooks) will be archived at the study site or in a centralized archive. Particular attention must be paid to the list identifying the patients included in the study and to the consent forms. This list and the consent forms are the most important documents in the files to be archived by the investigator.

All study-related documents must be kept for 15 years after the end of the study. At the end of this period, the Sponsor will inform the investigators of the end of archiving.

# STUDY FUNDING

## Protocol financing

This research project is funded by the Call for Research Projects - Psychoactive Substances and Addictive Behaviors SPA-CPA-V1, a joint initiative of INCA (Institut National du Cancer) and IReSP (Institut pour la Recherche en Santé Publique).

The budget will be managed by the Délégation à la Recherche Clinique et à l'Innovation, in agreement with the study's investigator-coordinator.

# REPORTS AND PUBLICATIONS

## Final report

The final study report will be written in collaboration with the study methodologists. It will be signed by the project coordinator and the sponsor, and sent to each principal investigator.

Any results of the study will be submitted for publication.

## Publication rules

The study will be reported to a registry meeting the specifications required for publication of its results in major international medical journals, as recommended by the ICMJE (*International Committee of Medical Journal Editors*).

All data collected in the course of this study are the property of the Study Sponsor and may not be communicated to a third party under any circumstances without the written consent of the Study Investigator.

The order of authors is defined as follows

*DEMINA Anastasia, XXX...*

Any publication or communication (oral or written) will be decided by mutual agreement between the investigators and will respect the international recommendations of the ICMJE: Recommendations for the conduct, reporting, editing, and publication of scholarly work in medical journals. International Committee of Medical Journal Editors; Updated December 2016

[www.icmje.org/icmje-recommendations.pdf](http://www.icmje.org/icmje-recommendations.pdf).

For any publication, the investigator must refer to :

- at CHU Dijon Bourgogne
- to the sponsor of the study as follows: "With the support of the Institut national du cancer and IReSP".

- the registration number in the Clinical Trial directory

In the case of publications produced jointly with the Université Bourgogne Franche Comté or any other research organization, the rules for addressing publications will comply with the terms of the agreement, i.e. :

- Université Bourgogne Franche-Comté, Inserm CAPS U1093, F-21000 Dijon, France
- CHU Dijon Bourgogne, Service hospitalo-universitaire d'addictologie, F-21000 Dijon, France
- Any other research organization and/or institution involved in the publication

# BIBLIOGRAPHY

1. CDCTobaccoFree. Health effects of smoking. Centers for Disease Control and Prevention. 2022

2. Bonaldi C. Estimation du nombre de décès attribuable au tabagisme, en france de 2000 à 2015 / estimation of deaths attributable to tobacco smoking, in france from 2000 to 2015. 2019;

3. Kulhánová I, Forman D, Vignat J, Espina C, Brenner H, Storm HH, et al. Tobacco-related cancers in Europe: The scale of the epidemic in 2018. Eur J Cancer. nov 2020;139:27-36.

4. WHO. Tobacco 2022

5. Hartmann-Boyce, Chepkin SC, Ye W, Bullen C, Lancaster T. Nicotine replacement therapy versus control for smoking cessation. Cochrane Tobacco Addiction Group, editor. Cochrane Database Syst Rev 31 May 2018 2019(1).

6. Goldberg SB, Rythme B, Griskaitis M, Willutzki R, Skoetz N, Thoenes S, et al. Mindfulness-based interventions for substance use disorders. Cochrane Drugs and Alcohol Group, editor. Cochrane Database Syst Rev. 20 Oct 2021 ;2021(10).

7. Khoury B, Lecomte T, Fortin G, Masse M, Therien P, Bouchard V, et al. Mindfulness-based therapy: A comprehensive meta-analysis. Clin Psychol Rev. August 2013;33(6):763-71.

8. Brasseur JA, Elwafi HM, Davis JH. Craving to stop smoking: psychological models and neurobiological mechanisms of mindfulness training as an addiction treatment. Transl Issues Psychol Sci. 2014;(S) :70-90.

9. Garrison KA, Pal P, O'Malley SS, Pittman BP, Gueorguieva R, Rojiani R, et al. Wanting to quit smoking: a randomized controlled trial of smartphone app-based mindfulness training for smoking cessation. Nicotine Tob Res Off J Soc Res Nicotine Tob. 16 2020;22(3):324-31.

10. Assessment of applications in the mobile health sector (mHealth) - Overview and quality criteria for medical content to reference digital services in the digital health space and professional services package. 2021;

11. Oikonomou MT, Arvanitis M, Sokolove RL. Mindfulness training for smoking cessation: a meta-analysis of randomized controlled trials. J Health Psychol. dec 2017 ;22(14) :1841-50.

12. Garland EL, Froeliger B, Howard MO. Mindfulness training targets neurocognitive mechanisms of addiction at the attention-evaluation-emotion interface. Frontal Psychiatry. 2014;

13. Grant S, Colaiaco B, Motala A, Shanman R, Booth M, Sorbero M, et al. Mindfulness-based Relapse Prevention for Substance Use Disorders: A Systematic Review and Meta-analysis. J Addict Med. oct 2017 ;11(5) :386-96.

14. Ruscio AC, Muench C, Brede E, Waters AJ. Effect of brief mindfulness practice on self-reported affect, craving, and smoking: a pilot randomized controlled trial using ecological momentary assessment. Nicotine Tob Res Off J Soc Res Nicotine Tob. jan 2016 ;18(1) :64-73.

15. Terhorst Y, Philippi P, Sander LB, Schultchen D, Paganini S, Bardus M, et al. Validation of the Mobile Application Rating Scale (MARS). PloS One. 2020;15(11):e0241480.

16. Piper ME, Bullen C, Krishnan-Sarin S, Rigotti NA, Steinberg ML, Streck JM, et al. Definition and measurement of abstinence in clinical trials of smoking cessation interventions: an updated review. Nicotine Tob Res Off J Soc Res Nicotine Tob. June 12, 2020;22(7):1098-106.

17. Baromètre santé 2010. Available at: https://www.santepubliquefrance.fr/etudes-et-enquetes/barometres-de-sante-publique-france/barometre-sante-2010

18. Hoover, E.B., Butaney, B., Bernard, K., et al. Comparing the Effectiveness of Virtual and In-Person Delivery of Mindfulness-Based Skills Within Healthcare Curriculums. Med.Sci.Educ. 32, 627-640 (2022). https://doi.org/10.1007/s40670-022-01554-5

19. Mrazek AJ, Mrazek MD, Cherolini CM, Cloughesy JN, Cynman DJ, Gougis LJ, Landry AP, Reese JV, Schooler JW. The future of mindfulness training is digital, and the future is now. Curr Opin Psychol. 2019 Aug;28:81-86. doi: 10.1016/j.copsyc.2018.11.012. Epub 2018 Nov 28. PMID: 30529975.

20 Stjernswärd S, Hansson L. Outcome of a web-based mindfulness intervention for families living with mental illness - A feasibility study. Inform Health Soc Care. 2017 Jan;42(1):97-108. doi: 10.1080/17538157.2016.1177533. Epub 2016 May 31. PMID: 27245198.,

21 Jennison V. Asuncion MA , Catherine S. Fichten PhD , Vittoria Ferraro MEd , Caroline Chwojka MA , Maria Barile MSW , Mai Nhu Nguyen BSc & Joan Wolforth EdD (2010) Multiple Perspectives on the Accessibility of E-Learning in Canadian Colleges and Universities, Assistive Technology, 22:4, 187-199, DOI: 10.1080/10400430903519944

TABLES OF APPENDICES

Appendix 1: Visual Analog Scale EVA.......................................................................39

Appendix 2: Mobile App Rating Scale (MARS-F) .........................................40

Appendix 1:

Echelle Visuel Analogique EVA

**Date: |__|__|/|__|__|/|__|__|__|__|**


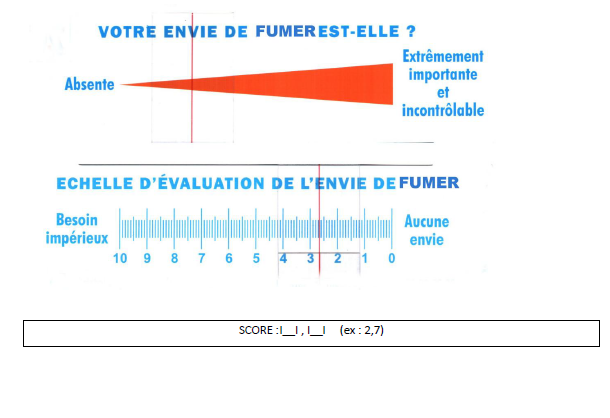


Appendix 2:

Mobile App Rating Scale, French version (MARS-F)

Classification of mobile applications

The Classification section is used to collect descriptive and technical information about the mobile application. Please consult the application description in iTunes / Google Play to access this information.

Mobile application name: __________________________________________________

Rating for this version: _________________ Rating for all versions: ______________

Développeur : _____________________________________________________________

Number of users who have rated this version: _________________________________

Number of users who evaluated all versions : ____________________________

Version: ____________________________ Last update: ___________________

Cost of basic version : _____________ Cost of updated version : ____________

Platform : ☐ iPhone ☐ iPad ☐ Android

Brief description of the mobile application: _______________________________________

_________________________________________________________________________

Target objectives of the application (several choices possible) :

☐ Happiness / Well-being

☐ Mindfulness / Meditation / Relaxation

☐ Negative emotions

☐ Depression

☐ Anxiety / stress

☐ Anger

☐ Behavior change

☐ Alcohol / substance use

☐ Personal challenge

☐ Entertainment

☐ Labor relations

☐ Physical health

☐ Other : _________________________

Theoretical context / Strategies used by the mobile application (several choices possible) :

☐ Evaluation

☐ Feedback

☐ Information / Education

☐ Monitoring / Follow-up

☐ Objective

☐ Tips / Tricks / Strategies / Skills training

☐ Cognitive behavioral therapy (CBT) - Behavior (positive events)

☐ Cognitive behavioral therapy (CBT) - Cognitive (thought stimulation)

☐ Commitment and acceptance therapy

☐ Mindfulness / Meditation

☐ Relaxation

☐ Recognition

☐ Based on strengths

☐ Other: _________________________

Affiliations :

☐ Unknown

☐ Commercial

☐ Government

☐ Non-governmental organization (NGO)

☐ University

Age group of target audience (multiple choices possible) :

☐ Children (under 12)

☐ Teenagers (13-17 years)

☐ Young adults (18-25)

☐ Adults

Technical aspects of the application (several choices possible) :

☐ Allow sharing (Facebook, Twitter, etc.)

☐ A community linked to the application

☐ Password protection

☐ Requires a login ID

☐ Send reminders

☐ Internet access required

Mobile application quality assessment

The scale assesses the quality of the application in 4 sections (A to D). All answers are rated on 5 modalities from "1. Inadequate" to "5. Excellent". Circle the answer that most accurately represents the quality of the application being evaluated. Please use the nomenclature provided for each response category.

SECTION A - Commitment

Fun, interesting, customizable, interactive (e.g., sends alerts, messages, reminders, feedback, allows sharing), targeted to a specific audience.

1. Entertainment: is the app fun/entertaining to use? Does it use strategies to increase engagement through entertainment (e.g. gamification)?

1. Dull, not fun or entertaining at all

2. Rather boring

3. OK, fun enough to entertain the user for a short time (<5 minutes)

4. Moderately fun and entertaining, will keep the user entertained for a while (5 to 10 minutes in total)

5. Very entertaining and fun, would stimulate repeated use

2. Interest: is the application interesting to use? Does it use strategies to increase engagement by presenting its content in an interesting way?

1. Not at all interesting

2. Rather uninteresting

3. OK, neither interesting nor uninteresting; would engage the user for a short time (<5 minutes)

4. Moderately interesting; would engage the user for some time (5 to 10 minutes in total)

5. Very interesting, would engage the user repeatedly

3. Customization: does the application provide or retain all the settings/preferences necessary for its functionality (e.g. sounds, content, notifications, etc.)?

1. Does not allow customization or requires a setting to be entered each time

2. Insufficient customization, limiting functionality

3. Allows basic customization for proper operation

4. Numerous customization options

5. Allows complete adaptation to user characteristics/preferences, retains all settings

4. Interactivity: does the application allow the user to intervene, provide feedback, contain invitations (reminders, sharing options, notifications, etc.)?

Note: to be perfect, these features must be customizable, not imposed.

1. No interactive functionality and/or no response to user interaction

2. Insufficient interactivity, or feedback, or user input options, limiting functionality

3. Basic interactive functions for proper operation

4. Offers a variety of interactive features / feedback / user input options

5. Very high level of responsiveness thanks to interactive features / feedback / user input options

5. Target group: Is the content of the application (visual information, language, design) adapted to the target audience?

1. Totally inappropriate / unclear / confusing

2. Generally inappropriate / unclear / confusing

3. Acceptable but not focused. Perhaps inappropriate / unclear / confusing

4. Well-targeted, with negligible problems

5. Perfectly targeted, no problems detected

Average score for section A - Commitment = ____

SECTION B - Functionality

Easy-to-learn application operation, navigation, flow logic, and gesture-based application design

6. Performance: how accurately / quickly do the application's functions and components (buttons / menus) work?

1. The application doesn't work; no response / insufficient response / imprecise response (e.g. crashes / bugs / broken functionality, etc.).

2. Some features work, but are slow or have major technical problems

3. The application works on the whole. Some technical problems need to be fixed / sometimes slow

4. Mainly functional with minor/negligible problems

5. Perfect response / fast; no technical bugs found / contains a "loading time remaining" indicator

7. Ease of use: how easy is it to learn how to use the application; how clear are the menu labels/icons and instructions?

1. No instructions / limited instructions; menu labels / icons are confusing; complicated

2. Usable after much time / effort

3. Usable after some time / effort

4. Easy to learn how to use (or clear instructions)

5. Able to use the application immediately; intuitive; easy

8. Navigation: is movement between screens logical / precise / appropriate / uninterrupted? Are all necessary navigation links between screens present?

1. Different sections of the application seem to have no logical sequence and are random / confusing / difficult to navigate

2. Can be used after considerable time and effort

3. Usable after some time / effort

4. Easy to use or lacks an insignificant link

5. Perfectly logical, simple, clear and intuitive navigation, fluidity or shortcuts.

9. Gesture design: are interactions (tapping / swiping / pinching / scrolling) consistent and intuitive across all components / screens?

1. Completely incoherent / confusing

2. Often inconsistent / confusing

3. OK with a few inconsistencies / confusing elements

4. Mainly consistent / intuitive with negligible problems

5. Perfectly coherent and intuitive

Average score for section B - Functionality = ____

SECTION C - Aesthetics

Graphic design, overall visual appeal, color scheme and stylistic consistency.

10. Layout: are the layout and size of buttons/icons/menus/content on the screen appropriate, or can they be zoomed in if necessary?

1. Very poor design, cluttered, some options impossible to select / locate / see / read, device display not optimized

2. Poor design, random, unclear, some options difficult to select/locate/view/read

3. Satisfactory, some problems with selecting/locating/viewing/reading items or minor screen size issues

4. Fairly clear, able to select/locate/view/read items

5. Professional, simple, clear, tidy, logically organized, optimized display on device. Every design element has a purpose.

11. Graphics: what is the quality / resolution of the graphics used for buttons / icons / menus / content?

1. Amateur graphics, very poor visual design - disproportionate, completely stylistically incoherent

2. Low-quality/low-resolution graphics; poor visual design - disproportionate, stylistically inconsistent

3. Graphics and visual design of average quality (generally consistent style)

4. High-quality graphics/resolution and visual design - predominantly proportioned, stylistically consistent

5. High-quality/resolution graphics and visual design - proportionate, stylistically consistent

12. Visual appeal: what is the quality of the application?

1. No visual appeal, unpleasant to look at, poorly designed, contrasting / mismatched colors

2. Low visual appeal - poorly designed, poor use of color, visually boring

3. Some visual appeal - average, neither pleasant nor unpleasant

4. High level of visual appeal - consistent graphics - coherent, professional design

5. As above + very attractive, memorable, striking; use of color enhances application functionality / menus

Average score for Section C - Aesthetics = ____

SECTION D - Information

Contains high-quality information (e.g. text, feedback, measurements, references) from a credible source. Select N/A if the question is not relevant.

13. Accuracy of application description (in the app store): does the application contain what is described?

1. Deceptive. The application does not contain the components/features described or has no description.

2. Imprecise. Application contains very few components / features described

3. OK. The application contains some of the components/features described below

4. Correct. The application contains most of the components/features described.

5. Very precise description of application components / functionalities

14. Objectives: Does the application have specific, measurable and achievable objectives (specified in the app store description or in the application itself)?

N/A The description does not list the objectives, or the objectives of the application are not relevant to the research objective (for example, using a game for educational purposes).

1. The application has no chance of achieving its objectives

2. The description lists certain objectives, but the application has very little chance of achieving them

3. Ok. The application has clear, achievable goals.

4. The application has clearly defined, measurable and achievable objectives

5. The application has specific, measurable objectives that are likely to be achieved

15. Quality of information: is the content of the application correct, well-written and relevant to the application's purpose/topic?

N/A There is no information in the application

1. Not relevant / inappropriate / inconsistent / incorrect

2. Poor. Very irrelevant / appropriate / consistent / may be incorrect

3. Moderately relevant / appropriate / consistent / and seems correct

4. Relevant / appropriate / consistent / correct

5. Very relevant, appropriate, coherent and correct

16. Amount of information: is the content in line with the application's stated objectives - comprehensive but concise?

N/A There is no information in the application

1. Minimal or overwhelming

2. Insufficient or possibly overwhelming

3. OK but not complete or concise

4. Offers a wide range of information, has some gaps or unnecessary details; or has no links to more information and resources

5. Comprehensive and concise; includes links to more information and resources

17. Visual information: is the visual explanation of concepts - through tables / graphs / images / videos, etc. - clear, logical, correct? - clear, logical, correct?

N/A There is no visual information in the application (for example, it contains only audio or text).

1. Not at all clear / confusing / wrong or necessary but missing

2. Generally unclear / confusing / wrong

3. OK but often unclear / confusing / wrong

4. Generally clear / logical / correct with negligible problems

5. Perfectly clear / logical / correct

18. Credibility: does the application come from a legitimate source (specified in the application store description or in the application itself)?

1. Source identified but legitimacy/reliability of source questionable (e.g. commercial enterprise with vested interest)

2. Appears to come from a legitimate source, but cannot be verified (e.g., has no web page)

3. Developed by an NGO / institution (hospital, etc.) / specialized commercial enterprise, funding agency

4. Developed by a government, university or as above but on a larger scale

5. Developed with nationally competitive government or research funding

19. Scientific evidence: has the application been tested/evaluated; needs to be verified by evidence (in published scientific literature)?

N/A Application not tested/evaluated

1. The evidence suggests that the application does not work.

2. The application has been tested (e.g. acceptability, usability, satisfaction rates) and has partially positive results in studies that are not randomized controlled trials (RCTs), or there is little or no contradictory evidence.

3. The application has been tested (e.g. acceptability, usability, satisfaction rates) and has positive results in studies that are not randomized controlled trials (RCTs), and there is no contradictory evidence.

4. The application has been tested and results evaluated in 1-2 randomized controlled trials (RCTs) showing positive results.

5. The application has been tested in over 3 high-quality randomized controlled trials (RCTs) with positive results.

Average score for section D - Information = ____

Subjective application quality

SECTION E

20. Would you recommend this application to others who could benefit from it?

1. Not at all, I wouldn't recommend this application to anyone.

2. There are very few people to whom I would recommend this application.

3. Perhaps there are several people to whom I would recommend it.

4. I would recommend this application to many people

5. I would definitely recommend this application to anyone.

21. How often do you think you would use this application over the next 12 months if it were relevant to you?

1. No

2. 1-2

3. 3-10

4. 10-50

5. >50

22. Would you pay for this application?

1. No

3. Maybe

5. Yes

23. What is your overall star rating for the application?

1. ✩ One of the worst applications I've used

2. ✩✩

3. ✩✩✩ Average

4. ✩✩✩✩

5. ✩✩✩✩✩ One of the best applications I've used

Average score for section E - Quality = ____

Evaluation summary :

Sections

A: Commitment Average score = ____

B: Functionality Average score = ____

C: Aesthetics Average score = ____

D: Information Average score = ____

E: Quality Average score = ____

Application quality : Average score = ____

Subjective application quality: Average score = ____

SECTION F - Application specifics

Raising awareness: this application is likely to raise awareness of the importance of the fight against smoking.

| Not at all  Agreed |  |  |  | Absolutely  Agreed |
| --- | --- | --- | --- | --- |
| 1⭘ | 2⭘ | 3⭘ | 4⭘ | 5⭘ |

Knowledge: this application is likely to improve knowledge/understanding of smoking.

| Not at all  Agreed |  |  |  | Absolutely  Agreed |
| --- | --- | --- | --- | --- |
| 1⭘ | 2⭘ | 3⭘ | 4⭘ | 5⭘ |

Attitudes: this application is likely to change attitudes towards improving smoking habits

| Not at all  Agreed |  |  |  | Absolutely  Agreed |
| --- | --- | --- | --- | --- |
| 1⭘ | 2⭘ | 3⭘ | 4⭘ | 5⭘ |

Intention to change: this application is likely to increase intentions/motivation to stop smoking.

| Not at all  Agreed |  |  |  | Absolutely  Agreed |
| --- | --- | --- | --- | --- |
| 1⭘ | 2⭘ | 3⭘ | 4⭘ | 5⭘ |

Help-seeking: the use of this application is likely to encourage people to seek further help for smoking (if necessary).

| Not at all  Agreed |  |  |  | Absolutely  Agreed |
| --- | --- | --- | --- | --- |
| 1⭘ | 2⭘ | 3⭘ | 4⭘ | 5⭘ |

Behavioral change: using this application is likely to reduce smoking

| Not at all  Agreed |  |  |  | Absolutely  Agreed |
| --- | --- | --- | --- | --- |
| 1⭘ | 2⭘ | 3⭘ | 4⭘ | 5⭘ |
