# Supplementary material for: Usability of a digital mindfulness training program for smoking cessation: A mixed-method single-center pilot study protocol (HowToMind)
Source: PLoS One. 2025 Feb 20;20(2):e0318686. doi: 10.1371/journal.pone.0318686 (PMC11841885; doi:10.1371/journal.pone.0318686)
Supplement: S1 File — (DOCX) [file pone.0318686.s002.docx]

| Information note **Usability of the digital mindfulness training program for smoking cessation: a mixed-method single-center pilot study protocol (HowToMind)**    2024-A00460-47 |
| --- |

##

## *(Done in 2 copies: one copy is given to the participant, the other is kept by the investigator)*

######

######

######

######

###### Sponsor: Dijon Bourgogne University Hospital – Delegation for Clinical Research and Innovation

###### 1, Bd Jeanne d'Arc

BP 77908 - 21079 Dijon Cedex

Tel: 03 80 29 50 15 / Fax: 03 80 29 36 90

Dear Madam/Sir,

The Investigating Physician

| Dr Anastasia DEMINA |
| --- |
| Name of the department: Addiction medicine |
| Name of the institution: CHU Dijon Bourgogne |
| Address: 14 rue Gaffarel 21079 DIJON |
| Phone number: 03.80.29.35.24 |

and the Dijon Bourgogne University Hospital are currently conducting a study on the usability of the digital mindfulness training program for smoking cessation in people who wish to quit smoking.

We would like to ask you to participate in this **interventional research with minimal risks and constraints**.

Here we present you with the information you need to understand the rationale and the timeline of the study, as well as the expected benefits, the constraints and the foreseeable risks of this study.

Your participation in this research is voluntary and you have the right to refuse to participate. You will continue to receive the best possible medical care, in accordance with current knowledge, even if you refuse to participate in this research.

**Read this document carefully, it belongs to you.**

**You can communicate it to your doctor or family for advice.**

**Ask any questions you think may be helpful.**

**Once you have received satisfactory answers to your questions and have been given an appropriate time to decide, you can then make the decision about your participation in this study.**

**OBJECTIVES AND METHODS OF THIS RESEARCH**

**Why this research?**

Several approaches are effective for smoking cessation. Despite this, relapses are frequent and the proportion of people who remain abstinent at 6 months is low (about 15-25%). New tools to make it easier to quit smoking are therefore needed.

Among these new tools, mindfulness-based interventions (MBI) are of interest. Indeed, there is already evidence of their effectiveness in smoking cessation. Usually, MBIs come in the form of 8-week programs, with weekly sessions conducted in groups. However, only a handful of patients have enough availability to attend the full program. In addition, these programs are only available to a small number of patients, due to the lack of trained professionals and out-of-pocket costs.

To deal with this, smartphone applications have been developed and evaluated, but none have demonstrated efficacy on smoking cessation. In addition, none of the apps evaluated offered the equivalent of a typical MBI in 8 weeks.

In our department, we are developing the first app-based MBI for smoking cessation that will allow you to follow a complete 8-week program. It recreates as faithfully as possible the conditions of a classic MBI. The content is created in our academic hospital addiction department by health professionals trained in mindfulness. We implemented the initial version of our program on a health data storage certified platform, proposed to us by the Regional Health Agency of Bourgogne - Franche Comté in partnership with the GRADeS (Regional Support Group for the Development of eHealth). Our electronic MBI is intended for clinical use in addition to standard treatment for smoking cessation.

According to the reference framework of good practices in eHealth, published by the French National Authority for Health in 2016 and updated in 2021, the evaluation of the acceptability and usability of an eHealth program by users is essential. We thus propose this pilot study to assess the acceptability and usability of the app, after 8 weeks of its use. The secondary objective is to study the impact of the app on tobacco consumption.

We expect good acceptability of the app with its continued use throughout the study. The knowledge of the acceptability and usability of our app, as well as the study of the motivations and obstacles to its use, will help us to make the necessary adjustments before deploying the app on a larger scale.

*Overview:*

HowToMind study is an interventional study that aims to study the usability and adherence to an app-based MBI in people who smoke who wish to stop their consumption.

- In this study, 60 participants are expected.

- The entire study will take place in Addiction medicine department at the Dijon Bourgogne University Hospital.

- The duration of learning Mindfulness using the app is 8 weeks. At the end of this period, you will be able to access all the resources of the app for an additional 4 weeks, to facilitate your autonomous practice. The objective is to achieve a practice of Mindfulness without any support.

- In addition to delivering a Mindfulness program, the app provides a smoking diary and the craving log. The app will also collect the frequency of your Mindfulness practice.

- All participants will receive standard treatment for smoking cessation, which consists of nicotine replacement therapy in the form of patches and lozenges as well as from specialized smoking cessation follow-up.

*How to participate in this study:*

During consultation in the Addiction medicine department at Dijon Bourgogne University Hospital, if you meet the eligibility criteria, the investigator will explain to you the contents of the present information note which will inform you about the nature of the research, its objectives, its methodology, its duration, the expected benefits, the foreseeable constraints and risks, according to article L1122-1 of the CSP.

**Inclusion visit:**

After carefully reading this information note, you will be able to give your oral consent to participate in the study and you will be given a copy of the information note and the Certificate of Obtaining Oral Consent.

During this visit, exhaled carbon monoxide (CO) will be measured and smoking consumption over the last 28 days will be evaluated. We will also assess the intensity of your cravings. Finally, we will present the app used in the study and give you an access code to a link to download the app.

The app contains 8 thematic modules related to tobacco addiction (one module per week). Key messages in the form of PDF documents and audio sessions ranging from 5 to 20 minutes each, will be available for daily practice (body scan, movement, breathing, etc.). The training modules will target specific elements of tobacco addiction, such as craving and automatic behavior, as well as more general elements such as emotion, self-judgment and acceptance.

Our app will allow you to gradually learn mindfulness, with a practice facilitated by recorded audio and video exercises. This learning phase will last eight weeks, then a self-directed practice is planned for the following 4 weeks. Thus, you will have access to all the resources of the app for a total of 12 weeks, with the objective of practicing mindfulness without support after 12 weeks of app-mediated practice.

This app also provides an interface accessible to the investigator to visualize the progress of the participants in the program and to manage alerts related to the questionnaires integrated into the app (questionnaires on the intensity of cravings, on the number of cigarettes consumed and the regularity of mindfulness practice).

**Follow-up visits:**

As part of this research, we will perform follow-up visits at 4 weeks (mid-program) and 8 weeks (end of program). During these visits, exhaled carbon monoxide (CO) will measured and smoking consumption will be assessed. We will also assess the intensity of your cravings. At the 8-week visit, you will be presented with a MARS usability scale to evaluate the ease of use of our app.

Between this last visit and the end of the study, we will offer you the opportunity to participate in a focus group (on a voluntary basis) in order to take into account your feedback on the app. We intend to adapt it following this collective and participatory work, so that it becomes as close as possible to users' expectations.

The last visit will be 12 weeks from the start of the study. During this visit, the measurement of exhaled carbon monoxide (CO) will be carried out and the assessment of smoking consumption will be made. We will also assess the intensity of your cravings.

**What are the possible benefits and risks?**

At the individual level, mindful practice, through learning the key messages of the MBI program during 8 weeks, could lead to better craving tolerance and a lower risk of relapse after smoking cessation.

At the collective level, the practice of mindfulness using our app could be an important addition to standard care to expand access to mindfulness to as many patients as possible.

MBIs have been extensively studied in various clinical trials in the field of mental health, addictions, pain, and stress management. The literature suggests that MBIs are well tolerated provided that contraindications (any acute mental or somatic condition) are respected.

Foreseeable side effects are those related to smoking cessation (cravings, difficulty concentrating, headaches, irritability, fatigue, hunger, constipation) and the use of nicotine replacement therapy (skin irritation, abdominal pain, hiccups, gastroesophageal reflux, sleep disturbances, nausea, diarrhea, dizziness).

**What are the modalities of medical care?**

In addition to the app, you will receive the treatment of reference in tobacco use disorder, which is a combination of patches and nicotine lozenges, and a specialized follow-up that can be continued beyond the duration of the study.

**CONDITIONS OF PARTICIPATION and TERMINATION OF PARTICIPATION IN THIS RESEARCH**

**To participate in this research**

•         You must be affiliated to national health insurance and not be a protected adult

•         For the duration of your participation, you must:

Inform the investigator of any medications you are taking and of any abnormal events;

 Follow the instructions given to you by the investigator or the research team.

•         You must not participate in any other research for the duration of the study (3 months).

**Termination of participation in this research**

All included individuals will be followed-up until the end of the study, except for patients who have withdrawn their consent

**Compensation**

If you participate in focus groups, at the end of your participation in the study, you will receive an indemnity of *thirty euros (€30)*as compensations for the constraints associated with the study.

In accordance with the current regulations, you are informed that the maximum amount of compensation received during a period of 12 months by a person participating in research is set at 6,000 Euros.

The payment of this compensation is linked to your compliance with all the constraints of the trial and to the fact that you carry out all the study visits and participate in focus groups. In the event of premature discharge due to consent withdrawal on your part or if the investigating physician decides to exclude you from the trial because you do not comply with the instructions given, you will not be compensated. However, in the event of premature discharge by medical decision of the investigating physician or for any other reason, you will be compensated proportionally.

**REGULATORY FRAMEWORK FOR THIS RESEARCH**

This study is carried out in accordance with Law No. 2012-300 of March 5, 2012, known as the "Jardé Law" relating to public health policy, amended by Ordinance No. 2016-800 of June 16, 2016 and its implementing decrees, and defined in 2° of Article L1121-1 of the Public Health Code.

The promoter complies with Article L.1121-10 of the Public Health Code and has taken out civil liability insurance guaranteeing its liability as well as that of any person involved in carrying out the research (Relyens company, contract number: 129.234, 18, rue Edouard Rochet, 69372 LYON Cedex 08).

In accordance with the regulations, this study received a favorable opinion for its implementation from the Ethics Committee (CPP Ile de France I) on April 16, 2024.

**Protecting your data**

This study will be carried out in accordance with Law No. 78-17 of 6 January 1978 relating to data processing, files and civil liberties, as amended, and European Regulation 2016/679 of 27 April 2016 on the Protection of Personal Data (GDPR), adopted at European level, and which came into force on 25 May 2018.

Your medical and personal data will be processed electronically in order to establish the results of the study, in accordance with the exceptions provided for in Article 9 of the GDPR allowing the processing of health data and to respond to a question of public interest in accordance with Article 6 of the GDPR.

Who will have access to your data for the research?

The information concerning your identity (surname, first name) will only be known to the medical team taking care of you as well as to the persons carrying out the quality control of the research mandated by the sponsor, by the health or control authorities, by the sponsor's data protection officer if you contact him [dpo@chu-dijon.fr] and, in the event of a dispute, by the authorized staff of the sponsor’s insurance organization.

These persons are subject to professional secrecy.

Your coded data will be accessible to the following people: the sponsor and persons acting on its behalf (data manager, statistician, methodologist, and pharmacovigilance), independent experts in charge of re-analyzing the data to verify the results of the research, with a view to their publication, under strict security conditions.

These persons, subject to professional secrecy, will have access to your coded data in the context of their function and in compliance with the regulations.

Data Retention Period, Reuse and Transfer

Your data will be kept for 15 years in accordance with Good Clinical Practice. These data may be used for future research conducted by the Dijon Bourgogne University Hospital. However, there are no plans to transfer personal data to another country.

What are your rights in relation to your personal data?

You have the right to access your data, through the investigator, and request that it be rectified or completed.

You can also request the restriction of the processing of your data (i.e. ask the sponsor to temporarily freeze the use of your data).

Even if you agree to participate in the research, you may at any time object to the processing of your data for the purposes of carrying out the research. In this case, no additional information about you will be collected.

You can also exercise your right to erasure of data already collected, but it may not be erased if this would seriously compromise the achievement of the research objectives.

In addition, certain data to ensure the quality and safety of the research (e.g. adverse effects) must be collected by the sponsor. You will not be able to exercise your right to object or to ask for erasure with regard to this data.

You can also access all of your medical data directly or through a doctor of your choice in accordance with the provisions of Article L. 1111-7 of the Public Health Code by contacting the investigator.

In the event of withdrawal of consent, the data previously collected will be used and may be processed under the conditions provided for by the research.

How can you exercise your rights?

You have several rights: the right to access and rectify data, the right to limit their computerized processing, the right to object to their transmission, the right to ask for deletion of data.

You can exercise your rights at any time and without having to give a reason.

As the sponsor does not have access to your identity, it is recommended that you first contact the investigator using the contact details available in this note.

In addition, if you wish, you can exercise your rights with the sponsor's Data Protection Officer [dpo@chu-dijon.fr] who will manage your request in coordination with the physician and professionals involved in the study. In this case, your identity (first name, last name) will be made accessible to the sponsor’s data protection officer.

You also have the right to file a complaint regarding the processing of your personal data with the Commission nationale de l'informatique et des libertés (CNIL), which is the supervisory authority competent in France data protection matters. You could do it either online: <https://www.cnil.fr/fr/plaintes> or by post to the following address: CNIL Service des Plaintes 3 place de Fontenoy TSA 80715 75334 Paris cedex 07.

**Your participation** **is** **voluntary**.

Your refusal to participate will have no impact on the type and quality of your care, as well as on your relationship with your doctor. If you agree to participate, you may leave this study at any time without justification or consequences on the quality of your care. All you need to do is inform your investigating physician.

**Thank you for your cooperation.**

**If you agree to participate in this study, we kindly ask you to give your oral consent.**

| Informed Consent **Usability of the digital mindfulness training program for smoking cessation: a mixed-method single-center pilot study protocol (HowToMind)**    2024-A00460-47 |
| --- |

*(Done in 2 copies: one copy is given to the participant, the other is kept by the investigator)*

**The Investigator of the Addiction medicine department suggested that I participate in the study HowToMind, of which the CHU DIJON BOURGOGNE is the sponsor.**

**I have been informed**of the objective and modalities of this research, the expected benefits, the foreseeable constraints and risks associated with this research as well as the conditions of my participation and my rights**.**

**I got the answers**to the questions I asked**.**

**I have read** the information note that has been explained to me and **I will keep a copy of it**.

**I declare** that I am affiliated to national health insurance.

For the reasons related to my safety and for the proper conduct of this research, **I accept to**:

-   answer any questions I may be asked about my medical history and follow all instructions given to me by the physician-investigator or his/her team, including those detailed in the information note;

-   contact the investigator or his/her team as soon as possible if I have an abnormal event.

**I also agree**:

-    That my entire medical file may be consulted by the persons authorized for this research;

-    With the collection of the medical and personal data described in the information note as well as their processing by the sponsor or by structures acting on its behalf;

**I noted that:**

-    The contact details of the medical investigator are available on the information note that was given to me;

-    My participation in this study is voluntary and I can decide at any time to interrupt my participation without justification and without affecting the quality of care I will receive.

-    I have rights relating to the protection of my personal data.

-    My consent does not relieve the investigator and sponsor of their responsibilities to me.

**I freely and voluntarily agree to participate in the research proposed to me.**

| Attestation of Obtaining Oral Consent from the Patient **Usability of the digital mindfulness training program for smoking cessation: a mixed-method single-center pilot study protocol (HowToMind)**    2024-A00460-47 |
| --- |

*(Done in 2 copies: one copy is given to the participant, the other is kept by the investigator)*

**I informed the patient** of the objective and methods of this research, the expected benefits, constraints and foreseeable risks associated with this research, as well as of the conditions of the patient’s participation and the patient’s rights.

**The patient read** the information note and received explanations along with a copy of the information note.

**The patient received the answers** to the questions asked.

| **Oral Consent**  Participant: ................................................................    Date of Oral Consent: *........./........./.........* | |
| --- | --- |
| **To be completed by the investigator**    I, ................................................................    **declare that I have obtained oral consent to participate in this research from the participant** | Signature              *........./........./..........* |
