# Supplementary material for: Usability of a digital mindfulness training program for smoking cessation: A mixed-method single-center pilot study protocol (HowToMind)
Source: PLoS One. 2025 Feb 20;20(2):e0318686. doi: 10.1371/journal.pone.0318686 (PMC11841885; doi:10.1371/journal.pone.0318686)
Supplement: S1 Appendix — (DOCX) [file pone.0318686.s003.docx]

Usability of the digital mindfulness training program for smoking cessation: a mixed-method single-center pilot study protocol

ClinicalTrials.gov: NCT06500117

| Data category | Information |
| --- | --- |
| Primary registry and trial identifying number | ClinicalTrials.gov, NCT06500117 |
| Date of registration in primary registry | 2024-07-08 |
| Secondary identifying numbers | NA |
| Source(s) of monetary or material support | INCA (French National Cancer Institute) and IReSP (Institute for Public Health Research) |
| Primary sponsor | CHU Dijon Bourgogne |
| Secondary sponsor(s) | None |
| Contact for public queries | AD anastasia.demina@chu-dijon.fr |
| Contact for scientific queries | AD anastasia.demina@chu-dijon.fr |
| Public title | Digital Mindfulness Training Program for Smoking Cessation and Maintenance (HowToMind) |
| Scientific title | Usability of the digital mindfulness training program for smoking cessation: a mixed-method single-center pilot study protocol |
| Countries of recruitment | France |
| Health condition(s) or problem(s) studied | Tobacco use disorder (DSM-5) |
| Intervention | eMind, digital mindfulness training program for smoking cessation in addition to the standard treatment (nicotine replacement therapy) |
| Key inclusion and exclusion criteria | Inclusion criteria: Adult smokers with tobacco use disorder motivated to stop smoking, with daily access to a smartphone, able to understand spoken and written French, who have given oral consent to participate |
|  | Exclusion criteria:  Persons under a legal protection measure or under court order  Individuals with cognitive problems that undermine mindfulness training  Individuals currently using smoking cessation treatments (burpropion, varenicline) except for NRT  Individuals with an acute psychiatric or somatic disorder requiring hospitalisation or not stabilised  Individuals with a contraindication to nicotine replacement therapy  Individuals with alcohol use disorder or using illicit substances  Individuals who are pregnant, breastfeeding or planning to become pregnant in the next 6 months  Individuals that are not affiliated to national health insurance |
| Study type | Single-center (Dijon, France) mixed-methods pilot study |
| Date of first enrolment | July 1st 2024 |
| Target sample size | 60 |
| Recruitment status | Recruiting |
| Primary outcome(s) | Acceptability and usability of digital mindfulness training program for smoking cessation |
| Key secondary outcomes | The change in smoking consumption and cigarette craving between baseline and 4 weeks, 8 weeks and 12 weeks |

Issue date: (initial protocol)

Authors: AD, ASF, BP, NMB, BT

**Sources and types of financial, material, and other support**

This research project is funded by the Call for Research Projects – Psychoactive Substances and Addictive Behaviours SPA-CPA-V1, a joint project between the INCA (National Cancer Institute) and the IReSP (Institute for Public Health Research). InCa and IReSP are public noncommercial healthcare organisms selecting protocols through calls for proposals with independent expert scientific committee evaluating methodological and scientific robustness of the proposed protocols. Funders had no role in the design of the study and they will not influence its implementation or execution, nor the analyses or interpretation/reporting of the obtained data.

Budget management will be carried out by the Clinical Research and Innovation Department of the Dijon Bourgogne University Hospital in agreement with the investigator-coordinator of the study.

The professionals of the addiction medicine department at the Dijon Bourgogne University Hospital, trained in both addiction medicine and mindfulness, created Mindfulness content that was implemented on a digital platform in the form of an e-health application called eMind. This implementation was made possible thanks to collaboration with public entities such as the Bourgogne Franche Comté Regional Health Agency (ARS BFC) and the Regional Group for the Development of e-Health (GRADeS). The latter have the Health Data Hosting (HDS) certification.

**Authors’ contributions**

AD is grant holder, conceived of the study, drafted the protocol, AD, ASF, NMB, BP, BT contributed to the study design and implementation. ASF provided statistical expertise. AD and NMB contributed to the qualitative analysis plan. All authors contributed to the manuscript and approved the final version.

**Trial Sponsor**: CHU Dijon Bourgogne

The Dijon Bourgogne University Hospital is the sponsor of this study. In accordance with the applicable regulations, the sponsor is responsible for:

- Registration of the study with the French National Agency for the Safety of Medicines and Health Products (ANSM)

- Information or application for authorization from ANSM

- Submission to the Ethics committee

- Declaration or request for authorization to the Commission Nationale Informatique et Liberté.

- Subscription to an insurance plan for interventional research defined in 1° and 2° of Article L1121-1 of the Public Health Code and in European Regulations EU 2017/745 and EU 536/2014

- Substantial changes: After the start of the study, any substantial protocol changes initiated by the investigator will be submitted to the sponsor; the latter must obtain, prior to its implementation, a favorable opinion from the CPP.

- Serious Breaches Statement on CTIS

- Statement of study onset defined by 1st inclusion

- Declaration of the end of study defined by the last patient's follow-up

###### Sponsor’s Reference: Dijon Bourgogne University Hospital – Delegation for Clinical Research and Innovation

**Composition, roles, and responsibilities of the coordinating center and committees**

***Principal investigator***

- Design and conduct of HTM

- Protocol preparation

- Organizing research committee meetings

- Preparation and publication of study reports

***Research department of Addiction medicine CHU Dijon Bourgogne***

- Investigators brochure preparation

- Recruitment of patients

- Data implementation

- Management of the patients’ appointments for follow-up

***Scientific committee (all authors are scientific committee members)***

- Design and conduct of HTM

- Protocol revisions

- CRF preparation and validation

- Evaluating progress of the study

- Agreeing on necessary amendments to the protocol if neede

***Trial management committee (principal investigator, administrator)***

- Study planning

- Legal aspects of implementation and conducting the trial

- Applying for ethics committee approval

- Responding to ethics committee inquiries

- Responding to funder’s inquiries

- Preparation of intermediate study reports

- Responsible for trial master file

- Budget administration

- Data verification

***Data manager***

- Data verification

- CRF implementation

- Maintenance of trial IT system and data entry
